# The role of focal adhesion kinase in bladder cancer: translation from *in vitro* to *ex vivo* human urothelial carcinomas

**DOI:** 10.2478/raon-2025-0052

**Published:** 2025-09-05

**Authors:** Gaja Markovic, Natasa Resnik, Aleksandar Janev, Dasa Zupancic, Gasper Grubelnik, Marusa Debeljak, Maja Cemazar, Tanja Jesenko, Masa Omerzel, Tomaz Smrkolj, Mateja Erdani Kreft

**Affiliations:** Institute of Cell Biology, Faculty of Medicine, University of Ljubljana, Ljubljana, Slovenia; Institute of Pathology, Faculty of Medicine, University of Ljubljana, Ljubljana, Slovenia; Clinical Institute for Special Laboratory Diagnostic, University Medical Center Ljubljana, Paediatric hospital, Ljubljana, Slovenia; Institute of Oncology Ljubljana, Ljubljana, Slovenia; Clinical Department of Urology, University Medical Center Ljubljana, Ljubljana, Slovenia; Department of Surgery, Faculty of Medicine, University of Ljubljana, Ljubljana, Slovenia

**Keywords:** human urothelial carcinoma, focal adhesion kinase, bladder cancer, urothelial cell, defactinib

## Abstract

**Background:**

Focal adhesion kinase (FAK), a cytoplasmic tyrosine kinase, plays a crucial role in focal adhesion turnover by interfacing between the extracellular space, transmembrane integrins, and actin filaments. Its significance for the progression of several malignancies, including bladder cancer, has been well-documented. However, its precise role and the implications of its inhibition in bladder cancer tissues and urothelial *in vitro* models has not been fully explored. This study examined FAK expression and function in human bladder cancer biopsies and *in vitro* bladder cancer models.

**Materials and methods:**

*Ex vivo* analyses were performed using reverse transcription-quantitative PCR (qRT-PCR), western blotting, and immunohistochemistry to compare FAK expression between bladder cancer tissues and adjacent normal tissues. *In vitro*, FAK expression was assessed in low-grade (LG) human non-invasive papilloma urothelial cell line RT4 for NMIBC (Ta), high-grade (HG) human muscle-invasive cancer urothelial cell line T24 for MIBC (T2) and normal porcine urothelial (NPU) cells using qRT-PCR and western blotting, as well as flow cytometry for the quantification of FAK-positive RT4 and T24 cells. The role of FAK in cancer cell survival was explored *in vitro* using microRNA (miRNA) to silence FAK expression. Additionally, we used FAK inhibitors PND-1186, PF-573228 and defactinib to investigate the effects of FAK inhibition on normal compared to cancerous bladder urothelial cells.

**Results:**

*Ex vivo* analyses demonstrated significantly higher FAK expression in bladder cancer tissues compared to adjacent normal tissues. Similarly, *in vitro* analyses showed significantly higher FAK expression in RT4 and T24 cells than NPU cells. Silencing FAK using anti-FAK plasmids led to increased caspase-3-mediated apoptosis of RT4 and T24 cells and growth reduction of stably transfected T24 cells. Importantly, based on cell viability assays, treatment with 100 μM defactinib for 2 hours per day on 3 consecutive days was identified as a clinically relevant regimen. Under this treatment, the viability of differentiated NPU cells remained high at 108.4 ± 17.1%, while the viability of 2-day RT4 and 2-day T24 cells was drastically reduced to 4.1 ± 2.7% and 7.6 ± 2.9%, respectively.

**Conclusions:**

To our knowledge, this is the first report demonstrating the role of FAK and its inhibition across both normal and cancerous bladder urothelial models. This study highlights the critical role of FAK in the progression of human bladder cancer and establishes a foundation for exploring FAK inhibition as a potential therapeutic approach in bladder cancer treatment.

## Introduction

Bladder cancer is the ninth most common cancer worldwide across genders and the sixth most common cancer in men, for whom it is also the ninth leading cause of cancer death.^1–3^ In Slovenia, the estimated incidence rate of bladder cancer (with 95% prediction interval) in 2024 was 27 (17–37) per 100,000 men and 11 (8–13) per 100,000 women.^4^ During the 2017–2021 period, the five-year net survival rates were 53.8% for men and 49.6% for women.^4^ Besides occupational exposure to chemicals and water pollution, cigarette smoking is the main risk factor for bladder cancer.^2,5^ Due to increasing life expectancy, the incidence and mortality of bladder cancer are expected to almost double in the future.^2,5^

The most common presenting sign of bladder cancer is painless haematuria. Patients may also experience frequent urination, urgency, nocturia and dysuria. Obstructive symptoms, such as reduced or intermittent urine stream, straining, and feeling of incomplete voiding may also be present if the tumour is near the bladder neck or urethra. Sings of metastatic disease typically relate to the most common metastasis sites, including the lymph nodes, bones, lungs, liver and peritoneum. Advanced or metastatic disease may present with general symptoms, such as fatigue and weight loss or with a palpable renal or bladder mass.^6^

The classification of bladder cancer follows the traditional tumour, node, metastasis (TNM) staging. If there are no nodal or distant metastases, the most important determination is the depth of tumour invasion (T stage). The main distinction is made based on the invasion of the tumour into or beyond the muscularis propria.^7^ Non-muscle-invasive bladder cancers (NMIBCs) are mainly papillary tumours (Ta and T1) that may invade subepithelial connective tissue, but do not invade the muscularis propria and occur in 75% of newly diagnosed patients.^5^ NMIBCs are typically treated with transurethral resection of bladder (TURB), followed by adjuvant intravesical therapy, which involves instillations of either chemotherapeutics or the Bacillus Calmette-Guérin vaccine. This approach aims to eliminate any residual cancer cells after tumour resection, thereby reducing the risk of disease recurrence and progression.^8,9^ Nevertheless, NMIBCs have a high recurrence rate (50–70%), which remains a critical obstacle to effective bladder cancer treatment. To address this challenge, various strategies are being developed to prolong drug residence time in the bladder and enhance drug penetration through the urothelium, most notably the use of electroporation-based therapy, mucoadhesive materials and nanocarriers.^9,10^ Intravesical instillations offer several advantages and are a promising area for therapeutic development. They offer easy access to the bladder via the urethra and the ability to achieve localised drug effects at high concentrations due to the low permeability of the urothelium.^9,11^ Additionally, the diminished differentiation of urothelial cancer cells compared to the highly differentiated normal urothelial cells presents an opportunity for targeted drug delivery. *In vitro* studies have shown that urothelial cancer cells internalise nanoparticles by increased endocytosis, while this is largely limited in normal urothelial cells.^10,11^

In contrast to NMIBC, cells of muscle-invasive bladder cancer (MIBC) invade the detrusor muscle (T2), perivesical fat (T3) or adjacent organs (T4) and often metastasise to regional lymph nodes (N1–N3) or distant organs (M1).^5^ MIBCs occur in 25% of newly diagnosed patients and are typically treated with radical cystectomy.^8^ Although disease-free survival after five years has increased thanks to perioperative chemotherapy, recurrence rates remain very high. This presents a significant challenge for treatment and underscores the need for the development of new therapeutic agents.^12^

One emerging potential therapeutic target is focal adhesion kinase (FAK), a cytoplasmic tyrosine kinase, which plays a significant role in the initiation and progression of several advanced-stage solid cancers, including bladder cancer.^13,14^ FAK is expressed in most tissues, where it regulates the turnover of focal adhesions by mediating interactions between extracellular matrix proteins, transmembrane integrins and actin filaments.^13,15^ The most extensively studied mechanism of FAK activation involves its dimerization, which follows integrin clustering upon binding to extracellular matrix proteins.^13^ This dimerisation leads to the autophosphorylation of FAK at Tyr-397, where SRC family kinases can bind and phosphorylate the FAK activation loop.^13^ The phosphorylated sites can subsequently bind to other molecules with SH2 domains, connecting phosphorylated FAK (p-FAK) to Ras activation and the MAPK pathway.^15^ The *PTK2* gene, which encodes FAK, is frequently amplified, and increased FAK mRNA levels have been observed in several cancers, including bladder cancer.^13^ Studies of tumour tissues have demonstrated that increased FAK expression and autophosphorylation at Tyr-397 are associated with tumour progression, reduced cell death, and increased metastatic potential via the increased invasiveness, proliferation, and migration of cancer cells.^13,16–19^

In this study, we aimed to investigate the role of FAK in *ex vivo* and *in vitro* models of bladder cancer, focusing particularly on the effects of FAK inhibition on normal urothelial cells compared to urothelial cancer cells, as most bladder cancers originate from the urothelium.^5^ We conducted molecular characterisation of several focal adhesion and adherens junctional proteins in *ex vivo* human bladder tissues and in different *in vitro* models. To evaluate different treatment modalities and the cytotoxic effects of FAK downregulation and inhibition, we employed gene electrotransfer of anti-FAK plasmids and treated *in vitro* models with the FAK inhibitors PND-1186, PF-573228 and defactinib.

## Materials and methods

### Cell cultures

Normal porcine urothelial (NPU) cells were established as previously described.^11,20,21^ In this study, we used both undifferentiated and differentiated *in vitro* models of NPU cells, which serve as a morphological and functional surrogate for normal human bladder urothelium. NPU cells were seeded at a density of 1 × 10^5^ viable cells/cm^2^ and grown for 2 days in culture medium containing equal amounts of MCDB153 (Sigma-Aldrich, St. Louis, MO, USA) and A-DMEM (Gibco, Life Technologies, Thermo Fisher Scientific, Waltham, MA, USA) supplemented with 15 μg/ml adenine (Sigma-Aldrich, St. Louis, MO, USA), 0.1 mM phosphoethanolamine (Sigma-Aldrich, St. Louis, MO, USA), 5 μg/ml insulin (Sigma-Aldrich, St. Louis, MO, USA), 0.5 μg/ml hydrocortisone (Sigma-Aldrich, St. Louis, MO, USA), 2 mM glutamax (Gibco, Thermo Fisher Scientific, Waltham, MA, USA), and 2.5% FBS (Invitrogen, Carlsbad, CA, USA) to establish the undifferentiated normal urothelial model (2-day NPU cells). To establish the differentiated normal urothelial model, NPU cells, after reaching confluence (at day 7), were cultured for another three weeks in the same culture medium as described above but without fetal bovine serum (FBS) and with 2.5 mM CaCl_2_. The differentiated normal urothelial model is a suitable *in vitro* model for investigating urothelial drug candidates.^20–23^ The preparation of primary urothelial cells from porcine urinary bladders was approved by the Veterinary Administration of the Slovenian Ministry of Agriculture and Forestry in accordance with the Animal Health Protection Act and the Instructions for Granting Permits for Animal Experiments for Scientific Purposes (Decree No. U34453-15/2013/2).

Other *in vitro* models include the low-grade (LG) human non-invasive papilloma urothelial cell line RT4 for NMIBC (Ta) and the high-grade (HG) human muscle-invasive cancer urothelial cell line T24 for MIBC (T2), both from ATCC (Manassas, VA). RT4 and T24 cells were seeded at a density of 1 × 10^5^ viable cells/cm^2^ and grown for 2 days in A-DMEM (Gibco) with Ham’s F12 (Gibco) to represent conditions early after tumour resection with few remaining urothelial cancer cells (2-day RT4 and 2-day T24 cells) or seeded at a density of 5 × 10^4^ viable cells/cm^2^ and grown for 7 days to represent the later stages of regeneration after tumour resection (7-day RT4 and 7-day T24 cells). The selected time points (2-day and 7-day models of RT4 and T24 cells) were chosen based on our previous studies indicating significant changes in cellular activity and biological processes at these time intervals. The 2-day models represent the early post-surgery phase, while the 7-day models represent later time points, where changes associated with tumour progression become more evident. This approach allows us to better understand the mechanisms involved in bladder cancer progression.

Normal urothelial and urothelial cancer *in vitro* models were cultured at 37°C and 5% CO_2_. All urothelial models were repeatedly tested negative for mycoplasma infection using MycoAlert mycoplasma detection kit (Lonza, Basel, Switzerland).

### Study design – participant recruitment

The study population consisted of 12 patients with either bladder cancer or normal urothelium who underwent TURB. Informed consent was obtained from all patients whose biopsies were included in this study. Tissue samples were collected from both cancerous and normal areas using cold cup biopsy, resulting in a total of 17 biopsies analysed. Participant recruitment and study design were at the discretion of the operating urologist, who determined the number of biopsy samples for each patient after intraoperative evaluation of tumour location, size, multiplicity, and the risk of acute bleeding. During a cold cup biopsy, a tissue sample is obtained using biopsy forceps. These forceps are at room temperature and feature two half-spherical jaws with sharp edges for sectioning. The forceps are introduced into the bladder via a cysto-scope. When the forceps close around the tumour tissue, a spherical tissue sample is cut between the jaws and retrieved by pulling the sample through the cystoscope. The diameter of the jaws, and thus the size of the tissue sample, is 4 mm. The biopsies captured the urothelium and part of the lamina propria. Samples were processed for western blot (from 9 patients) and paraffin embedding (from 8 patients). For paraffin sections, samples were fixed with 4% formaldehyde (FA) in phosphate buffered saline (PBS) overnight at 4°C and embedded in paraffin. Paraffin sections were 5 μm thick, cut from at least two different parts of each sample and stained with haematoxylin and eosin. The tissue samples were initially collected without prior knowledge of their nature. It was only after pathological evaluation that some samples were confirmed to represent normal urothelial tissue. Tissue samples were classified as normal if no signs of hyperplasia or dysplasia were detected. WHO classification of tumours of the urinary system was used for pathological staging and classification.^24^ Urothelial carcinomas were diagnosed as invasive papillary urothelial carcinoma low-grade with invasion into the lamina propria (pT1 LG), invasive papillary urothelial carcinoma high-grade with invasion into the lamina propria (pT1 HG) and invasive papillary urothelial carcinoma highgrade with invasion into the muscularis propria (pT2 HG). Among 12 patients, 10 (83%) were male (aged 62–79) and 2 (17%) were female (aged 80 and 98). Of the 10 male patients, 3 were diagnosed with pT1 LG, however, biopsies taken 1 cm posterior to the interureteric ridge revealed normal urothelium. The biopsy-based diagnoses for the male patients were therefore distributed as follows: pT1 LG (3 patients), pT1 HG (3 patients), and pT2 HG (3 patients). Among the 2 female patients, one was diagnosed with normal urothelium and the other one with pT1 LG.

### Quantitative reverse transcription polymerase chain reaction (qRT-PCR)

#### Isolation of total RNA

Isolation of total RNA from cell lines was performed using the AllPrep® DNA/RNA/miRNA Universal Kit (80224, Qiagen, Hilden, Germany), according to the manufacturer’s instructions and stored at -80°C. The concentration and quality of total RNA isolated was measured using the NanoDropTM 1000 (Thermo Fisher Scientific, Waltham, MA, USA).

#### Reverse transcription PCR

Prior to qRT-PCR, the RNA isolated from the cell cultures was diluted to a concentration of 180 ng of total RNA/μl. Reverse transcription was performed in a 10 μl reaction volume. 1 μl of Random Primer Mix (60 μM) (Invitrogen, Thermo Fisher Scientific, Waltham, MA, USA) was added to 3 μl of diluted RNA containing 540 ng of total RNA and incubated at 70°C for 5 min. After the first step, 1 μl of M-MuLV reaction mix (2×) and 5 μl of M-MuLV enzyme mix (10×) were added to each sample according to the protocol of the PCR programme, 25°C/5 min, 42°C/60 min, 80°C/4 min. The synthesised cDNA products were stored at -20°C.

#### Real time PCR

Prior to qRT-PCR, cDNA from cell cultures were diluted 20 times using RNAse-free water (2.7 ng cDNA/μl). For each qRT-PCR reaction, 3 μl of the diluted cDNA from the previous step was used (8.1 ng cDNA/reaction). 10 μl of PCR mix contained 5 μl PowerUpTM SYBR Green Master Mix (Applied Biosystems) and 1 μl of the forward and reverse primers (10 pmol) listed in Tables 1 and 2 *(GADPH*, Glyceraldehyde-3-Phosphate Dehydrogenase; *HPRT1*, Hypoxanthine Phosphoribosyltransferase 1; *PTK2*, Protein Tyrosine Kinase 2 (Focal Adhesion Kinase); *UP1B*, Uroplakin 1B; *UP3A*, Uroplakin 3A; *CDH1*, E-cadherin; *CDH2*, N-cadherin). PCR primers were designed according to the established laboratory protocol covering several exons of individual gene at the same region of the gene for *Homo sapiens* and *Sus scrofa*, nucleotide sequences are a bit different, but they amplify the same gene region. For the reference genes, *HPRT1* and *GAPDH* based on pretesting results were used. All PCR reactions were performed in triplicates. The PCR thermocycling procedure consisted of an initial step at 50°C for 2 min, followed by denaturation at 95°C for 2 min. This was followed by 40 cycles of annealing at 95°C for 15 sec and extension at 60°C for 60 sec. Afterward, a high-resolution melting analysis was conducted. The melt ramp ranged from 60°C to 95°C, with an initial conditioning before melting for 90 sec. The subsequent steps involved increasing the temperature by 0.7°C at each step, followed by a waiting period of 5 sec at each temperature.

**TABLE 1.** List of *Homo sapiens* primers used for reverse transcription polymerase chain reaction (qRT-PCR). Primers in bold are identical as used for *Sus scrofa* qRT-PCR

	Homo sapiens	
	Forward	Reverse
***GAPDH***	*TGCACCAACTGCTTAGC*	*GGCATGGACTGTGGTCATGAG*
***HPRT1***	***GGACTAATTATGGACAGGACTGA***	***CAGGTCAGCAAAGAATTTATAGC***
***PTK2***	*GGCCCAGAAGAAGGA*	*ATGCCTTGCTTTTCGCTGT*
***UP1B***	*AGCCTCTACCCACTGCTTGA*	***GGAAGAGGTTGGGTGTGAAA***
***UP3A***	*TCGTCATCACTTCCATCCTG*	*CGGACGTGTAGGAAGACTCC*
***CDH1***	*GAGGGGTTAAGCACAACAGC*	*AATGCCATCGTTGTTCACTG*
***CDH2***	*ACAGTGGCCACCTACAAAGG*	*CCGAGATGGGGTTGATAATG*

1 CDH1 = E-cadherin; CDH2 = N-cadherin; GADPH = glyceraldehyde-3-phosphate dehydrogenase; HPRT1 = hypoxanthine phosphoribosyltransferase 1; PTK2 = protein tyrosine kinase 2 (focal adhesion kinase); UP1B = uroplakin 1B; UP3A = uroplakin 3A

**TABLE 2.** List of *Sus scrofa* primers used for reverse transcription polymerase chain reaction (qRT-PCR). Primers in bold are identical as used for *Homo sapiens* qRT-PCR

	Sus scrofa	
	Forward	Reverse
***GAPDH***	*TGCACCACCAACTGCTTGGCA*	*GGCATGGACCGAGGTCATGAG*
***HPRT1***	***GGACTAATTATGGACAGGACTGA***	***CAGGTCAGCAAAGAATTTATAGC***
***PTK2***	*GCTGGATTATTTCGGTGGAG*	*CAGTAGCCGTCGATCAGGTC*
***UP1B***	*AGCCTCTACCCGCTGCTTGA*	***GGAAGAGGTTGGGTGTGAAA***
***UP3A***	*TCGTTATCACGTCCATCCTG*	*CAGACGTGTATGAAGGCTCC*
***CDH1***	*GACGGCTTAAGCACGACTGC*	*AACGCCTCCATTGCTTACTG*
***CDH2***	*AGTGGGATCCCCACCGCTGA*	*ATGGAAGGCAATCCCACGTA*

1 CDH1 = E-cadherin; CDH2 = N-cadherin; GADPH = glyceraldehyde-3-phosphate dehydrogenase; HPRT1 = hypoxanthine phosphoribosyltransferase 1; PTK2 = protein tyrosine kinase 2 (focal adhesion kinase); UP1B = uroplakin 1B; UP3A = Uroplakin 3A

### Western blot

Human samples were homogenised in ice-cold buffer (0.8 M Tris-HCl, 7.5% sodium dodecyl sulphate (SDS), 1 mM phenylmethylsulphonyl fluoride). Treated and untreated NPU, RT4 and T24 cells were scraped, pelleted and lysed in RIPA buffer (EMD Millipore, Darmstadt, Germany) supplemented with protease and phosphatase inhibitors (100× Halt Cocktail, Thermo Scientific, 78441). Protein concentration was determined using the BCA Protein Assay Kit (Thermo Fisher Scientific Waltham, MA, USA). 10 μg of proteins per lane were loaded onto 4–20% Novex Tris-Glycine gels (Thermo Fisher Scientific, Waltham, MA, USA), separated and transferred onto nitrocellulose membranes (Amersham Biosciences, Amersham, UK). The membranes were blocked in 5% non-fat dry milk in PBS supplemented with 0.1% Tween-20 (T-PBS) for one 1 h at room temperature, and then incubated overnight at 4°C with mouse monoclonal antibodies against E-cadherin (610182, 1:1,000, BD Transduction), rabbit polyclonal antibodies against N-cadherin (13116, 1:1,000, Cell Signaling), rabbit polyclonal antibodies against FAK (3285, 1:1,000, Cell Signaling), mouse monoclonal antibodies against p-FAK (sc-81493, 1:500, Santa Cruz), rabbit monoclonal antibodies against the heavy chain of p-FAK (70-025-5, 1:1,000, Thermo Fisher Scientific, Waltham, MA, USA) and rabbit polyclonal antibodies against 32 kDa proenzyme and the 17 kDa active form of caspase-3 (ab4051, 1:200, Abcam). To confirm equal protein loading, the blots were stripped with Restore Western Blot Stripping Buffer (Pierce, Rockford, IL) and reprobed with mouse monoclonal antibody against β-actin (A2228, 1:2,000, Sigma) or mouse monoclonal antibody against GAPDH (sc-47724, 1:1,000, Santa Cruz). HRP-conjugated secondary antibodies (anti-mouse and anti-rabbit IgG HRP-linked total antibodies, Sigma, 1:1,000) were used and detected with SuperSignal West Pico Plus chemiluminescent substrate (Thermo Fisher Scientific, Massachusetts, USA). Chemiluminescence signals were visualised using LAS-4000 CCD camera (Fujifilm, Tokyo, Japan) or iBright CL1500 (Thermo Fisher Scientific, Massachusetts, USA).

### Immunohistochemistry

Paraffin sections were deparaffinised and hydrated, and endogenous peroxidase activity was blocked with 3% H_2_O_2_ in methanol. Sections were heated in the microwave. Non-specific labelling was blocked with 5% bovine serum albumin (BSA). Sections were incubated overnight at 4°C with rabbit polyclonal antibody against FAK (3285, 1:100, Cell Signaling) and rabbit polyclonal antibody against p-FAK (70-025-5, 1:50, Thermo Fisher Scientific, Waltham, MA, USA). For negative controls, incubation with the primary antibody was omitted or the specific primary antibody was replaced by a non-relevant antibody. For secondary antibodies, biotinylated porcine anti-rabbit IgG (E353, 1:200, Dako) were applied for 1 h at room temperature, followed by incubation with ABC/HRP complex (Vector Laboratories, Burlingame, CA, USA). After the standard DAB (Sigma, Taufkirchen, Germany) development procedure, sections were counterstained with haematoxylin, and examined with a light microscope (Eclipse TE300, Nikon, Japan).

### Construction of anti-FAK plasmids

Three plasmid DNA encoding different miRNA against FAK (anti-FAK plasmids) were constructed using the pcDNATM6.2-GW/EmGFP-miR plasmid backbone (Invitrogen). For each plasmid two complementary single-stranded DNA oligonucleotides with the engineered pre-miRNA were used, precisely; containing a 4-nucleotide 5’ overhang complementary to the vector, followed by 5′G + 21-nucleotide pre-miRNA sequence (Table 3), a short 19-nucleotide spacer to form a terminal loop, a short sense target sequence with 2 nucleotides removed to create an internal loop and followed again by a 4-nucleotide 5’ overhang complementary to the vector.

**TABLE 3.** The anti-focal adhesion kinase (FAK) plasmids and their respective sense pre-miRNA sequences. Three plasmid DNA encoding different miRNA against FAK (anti-FAK plasmids) were constructed using the pcDNATM6.2-GW/EmGFP-miR plasmid backbone

Plasmid	Sense pre-miRNA sequence
p44	ATCTGTTTCTGACACAGAGAC
p45	AGAAATTTCTCTCTCACGCTG
p46	ATAGCAGGCCACATGCTTTAC

Standard molecular biology techniques of annealing, ligation and transformation were then performed in competent *E. coli* TOP10 (Thermo Fisher Scientific, Waltham, MA, USA) using the BLOCK-iT Pol II miR RNAi Expression Vector Kit with EmGFP (Thermo Fisher Scientific, Waltham, MA, USA). Expression of the miRNA molecules from the plasmid DNA in the cells was coupled to EmGFP expression under the control of the Pol II promoter, which allowed identification of the percentage of transfected cells with the plasmid DNA. Plasmid DNA was amplified in *E. coli* TOP10 and isolated using the EndoFree Plasmid Mega Kit (Qiagen, Hilden, Germany) according to the manufacturer’s instructions. The amount of isolated plasmid DNA was determined by a spectrophotometer at 260 nm (Epoch Microplate Spectrophotometer, BioTek, Winooski, VT, USA) and the quality was determined by measuring the ratio of absorbance at A260 nm/280 nm and by agarose gel electrophoresis. The working concentration of 1 mg/ml was prepared with endotoxin-free water. Plasmids were sequenced to confirm the presence, correct orientation and sequence of the double-stranded DNA oligonucleotide (ds oligo) insert.

### Gene electrotransfer (GET) of anti-FAK plasmids

RT4 and T24 cells were harvested when they reached approximately 80% confluence. A suspension of 25 × 10^6^ cells/ml was prepared in cold electroporation buffer (125 mM sucrose, 10 mM K_2_HPO_4_, 2.5 mM KH_2_PO_4_, 2 mM MgCl_2_×6H_2_O). 44 μL of the cell suspension was mixed with 11 μL of plasmids (1 mg/ml). Then, 50 μL of the suspension was electroporated using an electric pulse generator (GT-01, Faculty of Electrical Engineering, University of Ljubljana, Ljubljana, Slovenia). Six square pulses with a voltage/distance ratio of 1300 V/cm, a pulse duration of 100 μs and a frequency of 4 Hz were used, delivered via two parallel stainless-steel plate electrodes with a spacing of 1.9 mm. After GET, the cells were transferred to 24-well plates and incubated for 5 min before 1 ml of the appropriate culture medium was added. The cells were then grown until further analysis.

### FAK silencing efficiency and transfection efficiency

Cells were collected, and 1 × 10^6^ cells were prepared for staining. Cells were first washed twice in PBS and then 1 μL of FVD viability dye (eBioscience™ Fixable Viability Dye eFluor™ 780, 650865, Thermo Fisher Scientific) was added per 1 ml of cells and immediately vortexed. Cells were then incubated at 2–8°C for 30 min, protected from light and then washed twice more with PBS. After the last wash, the supernatant was discarded and samples were vortexed with a pulse vortex to completely dissociate the pellet. Cells were fixed by adding 100 μl of the Intracellular (IC) fixation buffer (eBioscience™, Thermo Fisher Scientific, Waltham, MA, USA), mixed and incubated again for 30 min at room temperature protected from light. 2 ml of 1× permeabilisation buffer (eBioscience™, Thermo Fisher Scientific, Waltham, MA, USA) was added and centrifuged at 400–600 × g for 5 min at room temperature. The supernatant was discarded, and this step was repeated once more. Cells were resuspended in 100 μl of 1× permeabilisation buffer and a 1:100 dilution of recombinant anti-FAK antibody [EP695Y] (ab40794, Abcam) was added and incubated for 30 min at room temperature, protected from light. Cells were then washed twice in PBS and incubated for 30 min in 100 μl of 1× permeabilisation buffer containing secondary Cy3 AffiniPure Donkey Anti-Rabbit IgG (H+L) (Jackson Immunoresearch, Ely, UK 711-165-152) antibody at a dilution of 1:100. 2 ml of 1× permeabilisation buffer was added and centrifuged at 400–600 × g for 5 min at room temperature. The supernatant was discarded, and this step was repeated once more. Stained cells were resuspended in 400 μl of IC fixation buffer and measured using the FACSCanto II flow cytometer (BD Biosciences, San Jose, CA, USA) using a 488-nm laser (air-cooled, 20 mW solid state) for excitation and both 530- and 650-nm bandpass filters were used for detection of green (GFP, transfection efficiency) and red (FAK silencing) fluorescence. To eliminate debris, cells were first gated and afterwards a histogram of gated cells against their fluorescence intensity was recorded. The number of fluorescent cells and their median fluorescence intensity were determined for each of the plasmids (software: BD FACSDiva V6.1.2).

### Determination of cell death

Mechanisms of cell death of RT4 and T24 cells were determined using the Annexin V (647) Apoptosis Detection Kit with 7-AAD (BioLegend, San Diego, CA, USA) according to the manufacturer’s instructions 24 h after GET of anti-FAK plasmids. Measurements were performed using the FACSCanto II flow cytometer (BD Biosciences, San Jose, CA, USA). A 488-nm laser (air-cooled, 20 mW solid state) was used for excitation and both 530- and 650-nm bandpass filters were used for green and red fluorescence detection. At least 40,000 events were measured for each cell sample. Data were analysed using BD FACSDiva software version 8.0.1 (BD Biosciences, San Jose, CA, USA). All debris was excluded for further analysis using the FSC/SSC scatter plot. The experiments were performed twice in three parallels. Live cells were identified as the subset of cells negative for both Annexin V and 7-AAD staining (Annexin V^–^/7-AAD^–^). Dead cells were defined as the subset negative for Annexin V and positive for 7-AAD staining (Annexin V^–^/7-AAD^+^). Apoptotic cells were determined as the sum of two subsets: early apoptotic cells, characterized by positive Annexin V and negative 7-AAD staining (Annexin V+/7-AAD^–^), and late apoptotic cells, characterized by dual positivity for Annexin V and 7-AAD (Annexin V+/7-AAD+).

### FAK inhibitors

**FIGURE 1.**
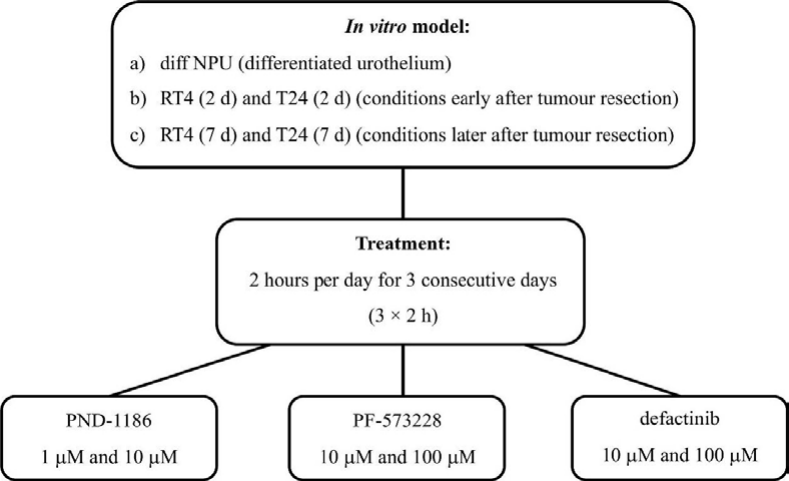
Schematic representation of focal adhesion kinase (FAK) inhibition. We used *in vitro* models of a) the primary model of differentiated normal urothelium (diff NPU), in which normal porcine urothelial (NPU) cells were maintained in culture for 4 weeks, 3 weeks of which were in medium UroM (+Ca^2+^ -S_FBS_), b) the bladder cancer urothelium early after resection of the bladder tumour, i.e. 2-day RT4 and 2-day T24 cells (RT4 (2 d) and T24 (2 d)), and of c) the bladder cancer urothelium late after resection of the bladder tumour, i.e. 7-day RT4 and 7-day T24 cells (RT4 (7 d) and T24 (7 d)). Cells were treated with FAK inhibitors PND-1186 (1 μM and 10 μM), PF-573228 (10 μM and 100 μM) and defactinib (10 μM and 100 μM) for 2 h per day for 3 consecutive days (3 × 2 h), after which cell viability was measured.

For the FAK inhibitor experiments, we selected three inhibitors, PND-1186, PF-573228, and defactinib, based on published efficacy data (all from SelleckChem, Houston, TX, USA). We prepared stock solutions following manufacturer’s instructions by dissolving the inhibitors in the solvent a-dimethylsulfoxide at room temperature (23°C). The stock solutions were then aliquoted into microcentrifuge tubes and stored at –80°C for up to six months. Before each experiment, we thawed the required number of tubes to room temperature. The stock solutions were then diluted in prewarmed growth medium to the desired final concentrations. For each inhibitor, we selected two concentrations: a lower concentration recommended for cell line experiments (1 μM for PND-1186, 10 μM for PF-573228 and defactinib) and a tenfold higher concentration to study and determine the maximum effect of the inhibitors. Any remaining stock solutions were stored in the refrigerator at 4°C for up to two weeks. The cells were treated as shown in Figure 1.

### Cell viability assay

Prior to the experiments, cells were seeded on black 96-well polystyrene plates (Costar, Kennebunk, ME, USA) as described above. Subsequently, the cells were treated with FAK inhibitors for 2 h per day for 3 consecutive days, which we labelled 1 × 2 h for the first day, 2 × 2 h for the second day and 3 × 2 h for the third day. The culture medium containing FAK inhibitors was replaced every 24 h. At the end of the 3-day treatment, cell viability was determined using the CellTiter-Glo Luminescent Cell Viability Assay (Promega, Madison, WI, USA) according to the manufacturer’s instructions. Luminescence was measured using a microplate reader (Safire2, Tecan, Mannedorf, Switzerland). Experiments were carried out in triplicate across at least three independent repetitions. For each cell line the data were presented as a percentage of the cell viability, calculated according to the following formula:

$$\;Cell\;viability\;=\frac{\text{ Average luminiscence intensity of treated cells }}{\text{ Average luminiscence intensity of untreated cells }}x100\%$$

### Immunolabelling

Cells grown on glass coverslips were untreated or treated with FAK inhibitors. Then the cells were fixed with 4% FA (in PBS) for 10 min at room temperature. The fixed cells were washed in PBS and then incubated with blocking solution (0.5% bovine serum albumin, 0.1% saponin, 0.1% gelatin, 50 mM NH_4_Cl, 0.02% NaN_3_) for 30 min at 37°C. After incubation, cells were washed in PBS and incubated overnight at 4°C with primary antibodies. We used the following primary antibodies: FAK (rabbit polyclonal antibody, 3285, 1:50, Cell Signaling), p-FAK (Tyr-397) (rabbit monoclonal antibody, 700255; 1:250, Thermo Fisher Scientific, Waltham, MA, USA) and cleaved caspase-3 (rabbit polyclonal antibody, ab2302, 1:100, Abcam). Cells were then washed in PBS and incubated for 90 min at 37°C with secondary goat anti-rabbit Alexa Flour555 antibody (1:500, Thermo Fisher Scientific, Waltham, MA, USA). To label actin, cells were then washed in PBS and additionally fixed for 15 min at 37°C in 4% FA. The cells were then incubated with 16.7 μg/ml phalloidin-FITC (Sigma) for 1 h at room temperature. Cells were then washed in PBS and embedded in Vectashield with DAPI (Vector Laboratories). Imaging was performed using the Axio Imager Z.1 fluorescence microscope (Zeiss, Germany).

### Statistical analyses

Cell viability values were statistically analysed using SPSS (version 23; IBM Corporation, USA). The data are average values from at least three cell viability experiments, each performed in a triplicate. Levene’s test was used to test for homogeneity of variances. If the variances were homogeneous, ANOVA, followed by Tukey’s post-hoc test was performed to determine statistical significance. If the variances were not homogeneous, Welch’s test was performed followed by Games-Howell’s post-hoc test. When comparing a pair of data sets, the twotailed Student’s t-test was performed. Differences were considered statistically significant at P < 0.05.

Relative quantification of gene expression was calculated using the 2^−ΔΔCt^ method.^25^ The reference GAPDH was used as an endogenous control for normalisation of the data. SPSS (version 24; IBM Corporation, USA) was used for statistical analyses. Changes in relative gene expression profiles between different cell lines were assessed using the Mann-Whitney U test where appropriate. In all cases, P ≤ 0.05 was considered to indicate a statistically significant difference.

### Ethics approval and consent to participate

The study was conducted in accordance with the Declaration of Helsinki and approved by the National Medical Ethics Committee of the Ministry of Health of the Republic of Slovenia under approval number 76/10/10. Informed consent was obtained from all patients whose biopsies were used in this study.

## Results

### Bladder cancer tissue exhibits a higher expression of focal adhesion kinase and adherens junctional proteins compared to normal bladder tissue

**FIGURE 2.**
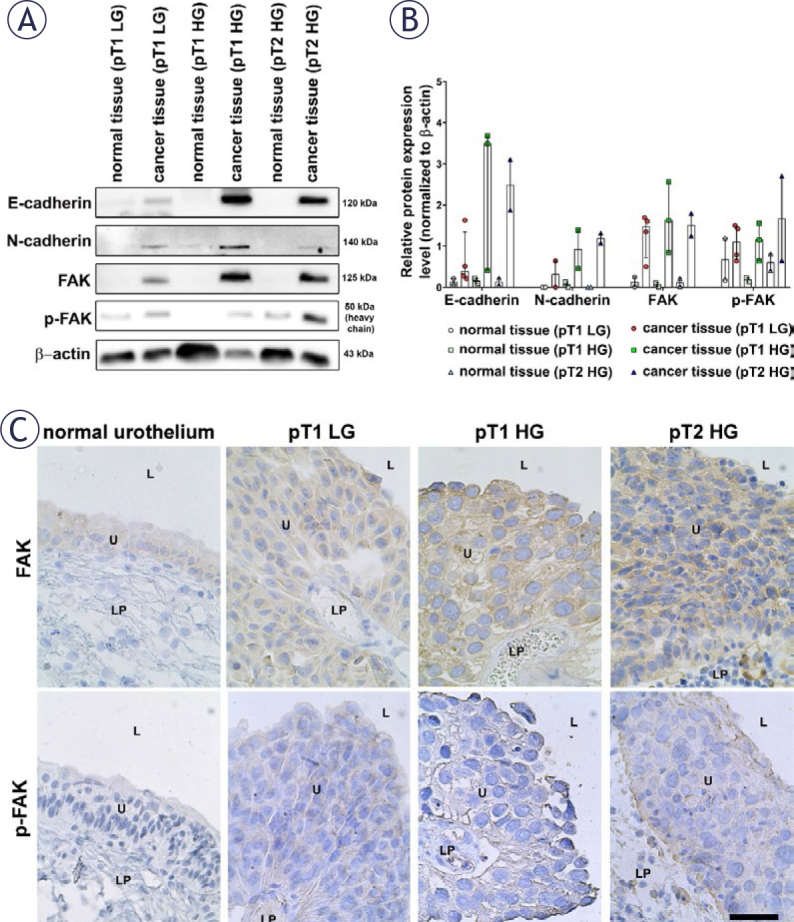
Western blot and immunohistochemistry analysis of *ex vivo* human bladder cancer samples. **(A)** Western blot analysis showing the relative expression levels of FAK, p-FAK, E-cadherin, and N-cadherin in pT1 low-grade (LG) and highgrade (HG) cancer tissues, pT2 HG cancer tissues, and the corresponding normal tissues (labelled as “normal tissue (pT1 LG)” for example), collected from the tumour-adjacent regions, which were not deemed normal at biopsy, but only after pathological evaluation. **(B**) Relative protein expression levels from biopsies of 2–4 patients ± the standard error of the mean (SEM) normalised to β-actin and presented as median with interquartile range. **(C)** Immunohistochemistry of FAK and p-FAK in normal tissue, pT1 LG and HG cancer tissues and pT2 HG cancer tissues. FAK = focal adhesion kinase; L = lumen; LP = lamina propria; U = urothelium. Scale bar: 50 μm.

To gain insights into the molecular characteristics of normal bladder tissue and bladder cancer tissues, we performed a comprehensive analysis of protein expression in *ex vivo* samples of human bladder cancer of varying grades and stages. In particular, we focused on studying the expression of E-cadherin, N-cadherin, FAK and p-FAK. Our results showed that their expression levels were higher in bladder cancer tissues compared to normal bladder tissues, which was confirmed by western blot analysis (Figures 2A, B and Supplementary Figure 1). Specifically, we observed the highest expression levels of E-cadherin and N-cadherin in pT1 HG and pT2 HG bladder cancer tissues, while the expression levels of FAK and p-FAK remained consistent across different grades and stages of bladder cancer tissue and were higher than in normal bladder tissue (Figures 2A, B and Supplementary Figure 1). In addition, immunohistochemistry showed a prevalence of FAK and p-FAK in pT1 LG, pT1 HG and pT2 HG bladder cancer tissue compared to adjacent normal bladder tissue and not in the lamina propria (Figure 2C).

### Urothelial cancer cells RT4 and T24 exhibit higher expression levels of FAK compared to normal urothelial cells NPU

**FIGURE 3.**
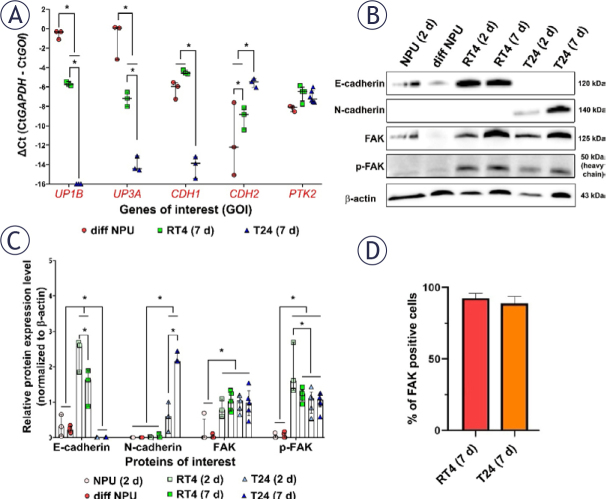
Molecular characterisation of normal urothelial cells (NPU) and bladder cancer cells (RT4 and T24) *in vitro*.**(A)** Relative expression levels of *UPK1B, UPK3A, CDH1, CDH2* and *PTK2* were determined using qRT-PCR in differentiated NPU cells (diff NPU), 7-day RT4 and 7-day T24 cells (RT4 (7 d) and T24 (7 d)). **(B)** Expression of E-cadherin, N-cadherin, FAK and p-FAK in 2-day NPU cells (NPU (2 d)), diff NPU, 2-day RT4 cells (RT4 (2 d)), RT4 (7 d), 2-day T24 cells (T24 (2 d)) and T24 (7 d), determined by western blot. **(C)** The relative protein expression of E-cadherin, N-cadherin, FAK and p-FAK in NPU (2 d), diff NPU, RT4 (2 d), RT4 (7 d), T24 (2 d) and T24 (7 d) normalised to the expression of β-actin. The results are presented as median with interquartile range. **(D)** Quantification of FAK-positive RT4 (7 d) and T24 (7 d) by flow cytometry. Data are presented as the mean ± the standard error of the mean (SEM). *P < 0.05.

In our study, we examined the relative expression levels of genes *UPK1B* (uroplakin 1b), *UPK3A* (uroplakin 3a), *CDH1* (E-cadherin), *CDH2* (N-cadherin) and *PTK2* (FAK) in different *in vitro* models of urothelial cells (Figure 3). Notably, the differentiated urothelial cells, obtained by maintaining NPU cells in culture for 4 weeks (3 weeks of which in UroM medium with Ca^2+^ and without FBS) represented normal human bladder urothelium, while the 2-day RT4 and 2-day T24 cells represented an early post-tumour resection state with few residual cancer cells. The 7-day RT4 and 7-day T24 cells represented later stages after tumour resection. These models were used in subsequent experiments to investigate the effects of FAK silencing and inhibition on the survival of normal urothelial and urothelial cancer cells.

The relative expression levels of *UPK1B* and *UPK3A* were significantly higher in differentiated NPU cells than in 7-day RT4 and 7-day T24 cells (Figure 3A). In contrast, the expression level of *CDH1* was higher in 7-day RT4 and differentiated NPU cells, while no expression was detected in 7-day T24 cells. Conversely, 7-day T24 cells had a significantly higher expression level of *CDH2* than 7-day RT4 and differentiated NPU cells. *PTK2* expression was not significantly different among differentiated NPU, RT4 and T24 cells.

In addition, we performed western blot analysis to determine the relative protein expression levels of E-cadherin, N-cadherin, FAK and p-FAK in the *in vitro* models (Figures 3B, C and Supplementary Figure 2). The relative protein expression level of E-cadherin was significantly higher in 2-day RT4 than in 7-day RT4 cells, and both had significantly higher protein expression levels of E-cadherin than NPU and T24 cells, regardless of the number of cultivation days (Figure 3C). Notably, 2- and 7-day T24 cells did not express E-cadherin but showed a significantly higher protein expression level of N-cadherin than 2-day NPU, differentiated NPU, 2-day RT4 and 7-day RT4 cells. 7-day T24 cells showed significantly higher levels of N-cadherin than 2-day T24 cells. In addition, the expression of FAK and p-FAK was significantly higher in RT4 and T24 cells compared to NPU cells (Figures 3B, 3C and Supplementary Figure 2).

To validate the western blot data on FAK expression in urothelial cancer cell lines RT4 and T24, we quantified the percentage of FAK-positive cells by flow cytometry. Our results showed no significant differences in FAK expression between 7-day RT4 and 7-day T24 cells (Figures 3D and Supplementary Figures 3-6).

### FAK silencing leads to apoptosis and necrosis of urothelial cancer cells RT4 and T24

**FIGURE 4.**
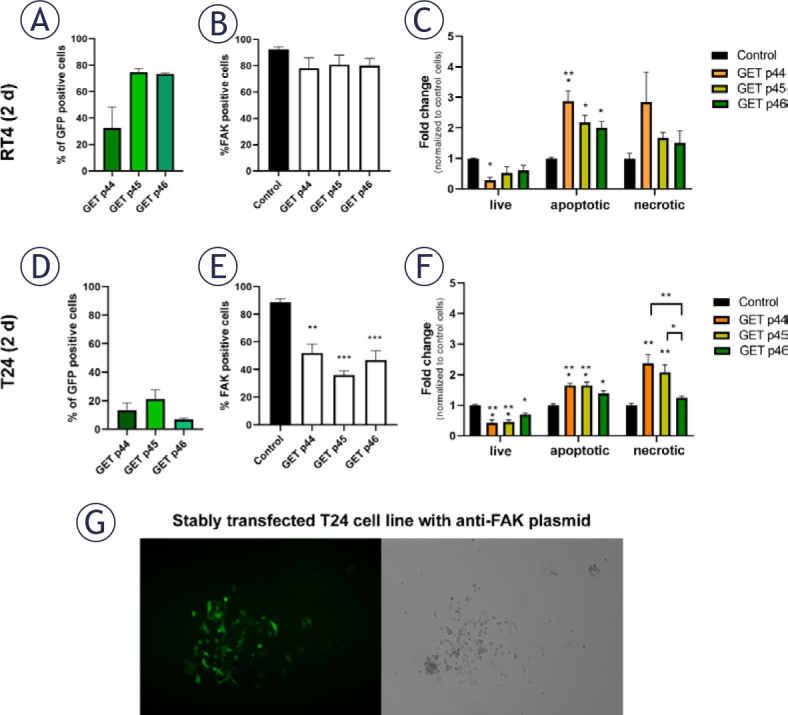
Effect of focal adhesion kinase (FAK) silencing on the survival of 2-day RT4 and 2-day T24 cells (RT4 (2 d) and T24 (2 d)). **(A–C)** Gene electrotransfer (GET) of anti-FAK plasmids p44, p45 and p46 in RT4 (2 d) did not result in statistically significant FAK silencing in RT4 cells but did result in their apoptosis. **(D–F)** GET of anti-FAK plasmids p44, p45 and p46 caused statistically significant FAK silencing and led to apoptosis of T24 (2 d). Plasmids p44 and p45 caused a statistically significant increase in necrosis of T24 (2 d). *P < 0.05, **P < 0.001. **(G)** T24 cells were stably transfected with anti-FAK plasmid p45 after 14 days. However, growth of these cells was limited to a single colony, as seen under fluorescence and phase contrast microscope. Data are presented as the mean ± the standard error of the mean (SEM).

To investigate the role of FAK in urothelial cancer cells, we performed gene electrotransfer (GET) of three anti-FAK plasmids to induce FAK silencing in 2-day RT4 and 2-day T24 cells (Figure 4). Plasmids p44, p45 and p46 encode both anti-FAK miRNA and green fluorescent protein (GFP) mR-NA, which share the same promoter region. All anti-FAK plasmids were successfully transfected into RT4 and T24 cells, as shown by the GFP-positive cells (Figures 4A, D). FAK silencing was not significantly different for all plasmids tested in 2-day RT4 cells and was only about 10% lower than that of the control (Figure 4B). However, all three plasmids induced a significant increase in apoptosis, but not necrosis, in 2-day RT4 cells (Figure 4C). All three plasmids induced a statistically significant silencing of FAK in 2-day T24 cells, especially plasmid p45 (Figure 4E). All three anti-FAK plasmids caused a significant increase in apoptosis of 2-day T24 cells, whereas only plasmids p44 and p45 caused a statistically significant increase in necrosis (Figure 4F).

To achieve stable silencing of FAK, a stably transfected T24 cell line was produced using GET with the anti-FAK plasmid p45. The growth of these cells was inhibited and limited to the size of a colony that declined after 14 days, demonstrating a crucial role of FAK in the growth and survival of T24 cells (Figure 4G).

### Defactinib-mediated FAK inhibition selectively reduces the viability of urothelial cancer cells RT4 and T24, while sparing normal urothelial cells NPU

The effects of FAK inhibition on normal urothelial and urothelial cancer cells have not been thoroughly investigated. We analysed the role of FAK inhibition on the survival of normal urothelial and urothelial cancer cells with the aim of determining a possible treatment modality in the form of intravesical instillations after bladder tumour resection.

**FIGURE 5.**
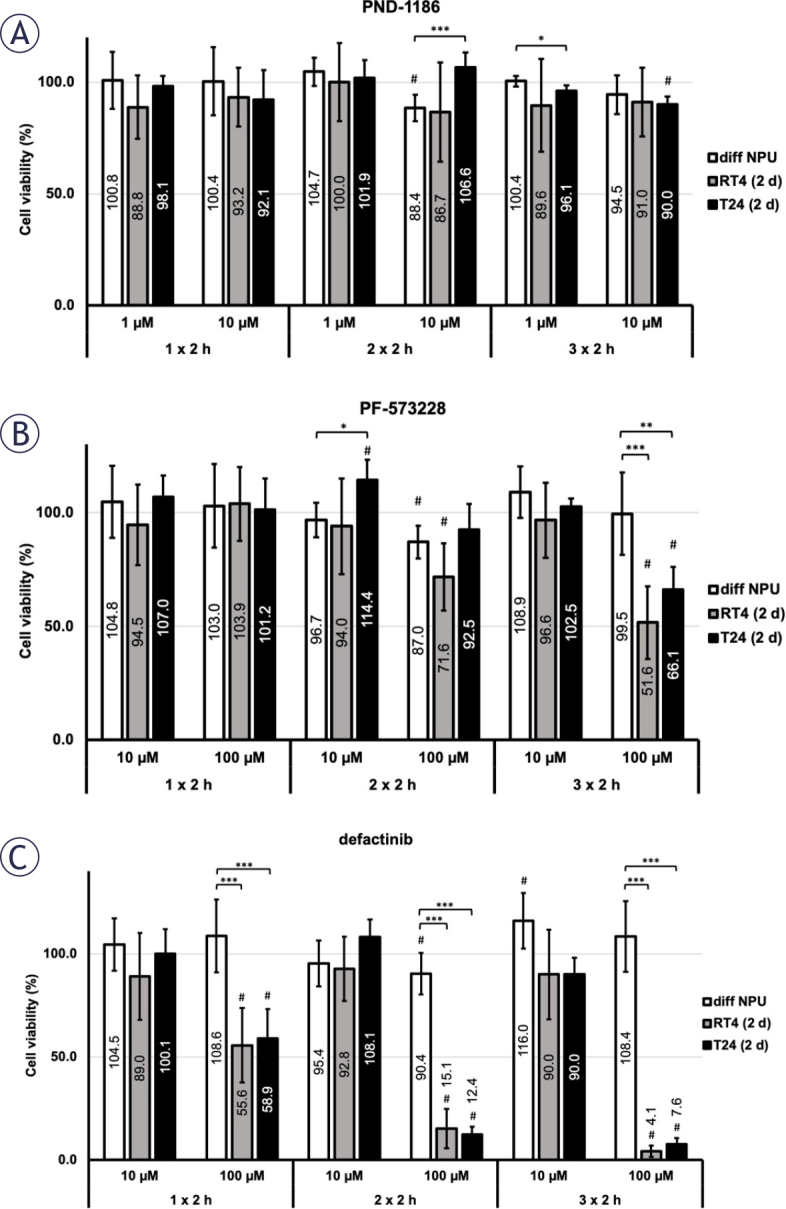
Effect of focal adhesion kinase (FAK) inhibitors on viabilities of differentiated normal urothelial (NPU) cells (diff NPU), 2-day RT4 and 2-day T24 cells (RT4 (2 d) and T24 (2 d)). **(A)** Treatment with FAK inhibitor 1 μM PND-1186 for 2 h per day for 3 consecutive days (3 × 2 h) led to a significantly lower viability of T24 (2 d) than diff NPU. **(B)** After 3 × 2 h treatment with 100 μM PF-573228, the viability of diff NPU was significantly higher than the viabilities of RT4 (2 d) and T24 (2 d). **(C)** Treatment with 100 μM defactinib for 3 × 2 h resulted in the greatest decrease in the viabilities of RT4 (2 d) and T24 (2 d) compared to the viability of diff NPU. Data are presented as the mean ± the standard error of the mean (SEM). To differentiate the statistical analysis, we compare the viability between different in vitro models (*, **, ***) and the viability between the control and the treated sample within each cell type (#). #P < 0.05. *P < 0.05. **P < 0.005. ***P < 0.001.

We measured the viabilities of different *in vitro* models following treatment with the FAK inhibitors PND-1186 (1 μM and 10 μM), PF-573228 (10 μM and 100 μM) and defactinib (10 μM and 100 μM). Incubation with 1 μM PND-1186 caused a significantly lower viability of 2-day T24 cells (96.1 ± 2.6%) compared to differentiated NPU cells (100.4 ± 2.3%) after treatment for 2 h per day for 3 consecutive days (3 × 2 h). Incubation with 10 μM PND-1186 for 3 × 2 h resulted in a greater decrease in the viabilities of both cancer cells lines (91.0 ± 15.4% for 2-day RT4 and 90.0 ± 3.6% for 2-day T24 cells) compared with differentiated NPU cells (94.5 ± 8.7%), but the difference was not significant. The decrease in the viability was also statistically significant for 2-day T24 cells after the 3 × 2 h treatment compared to T24 control cells (Figure 5A). Treatment with PND-1186 for 1 × 2 h did not lead to a significant decrease in the viabilities of any cells treated with inhibitors compared to their respective controls, nor among the different *in vitro* models.

Incubation with 10 μM PF-573228 only caused a significant difference between the viabilities of 2-day T24 cells (114.4 ± 9.0%) and differentiated NPU cells (96.7 ± 7.7%) after treatment for 2 × 2 h. On the other hand, treatment with 100 μM PF-573228 for 3 × 2 h resulted in significantly lower viabilities of both 2-day RT4 and 2-day T24 (51.6 ± 16.1% and 66.1 ± 9.9%, respectively) compared to differentiated NPU cels (99.5 ± 18.1%) (Figure 5B). Treatment with 100 μM PF-573228 for 2 × 2 h resulted in a significant decrease in the viability of differentiated NPU and 2-day RT4 cells compared to their respective controls (87.0 ± 7.1% and 71.6 ± 14.9%, respectively). Similarly, treatment with PF-573228 for 1 × 2 h did not result in significant differences in the reduction of cell viabilities compared to controls, nor among the cell viabilities of the different *in vitro* models.

Treatment with 10 μM defactinib did not lead to a significant difference between the viabilities of any of the different *in vitro* models. On the other hand, treatment with 100 μM defactinib significantly reduced the viability of urothelial cancer cells, while the viability of normal urothelial cells was unaffected (Figure 5C). It caused statistically significant differences between the viabilities of normal urothelial compared to urothelial cancer cells at each treatment interval. The 1 × 2 h treatment caused a statistically significant difference between the viabilities of differentiated NPU (108.6 ± 17.7%) compared to 2-day RT4 and 2-day T24 cells (55.6 ± 18.0% and 58.9 ± 14.2%, respectively). The 2 × 2 h treatment caused an even greater decrease in the viabilities of 2-day RT4 and 2-day T24 (15.1 ± 9.6% and 12.4 ± 3.9%, respectively) compared to differentiated NPU cells (90.4 ± 10.1%). Our study showed that differentiated NPU cells remained highly viable after FAK inhibition with 100 μM defactinib for 3 × 2 h (108.4 ± 17.1%), whereas 2-day RT4 and 2-day T24 cells were almost completely eliminated with viabilities of 4.1 ± 2.7% and 7.6 ± 2.9%, respectively. These findings could pave the way for clinical applications, such as a treatment involving the instillation of defactinib into the bladder for 2 h at a time. Such adjuvant therapy could be beneficial in destroying the residual urothelial cancer cells from papillary and muscle invasive carcinomas after resection of the bladder tumour, while leaving normal urothelial cells unaffected.

**FIGURE 6.**
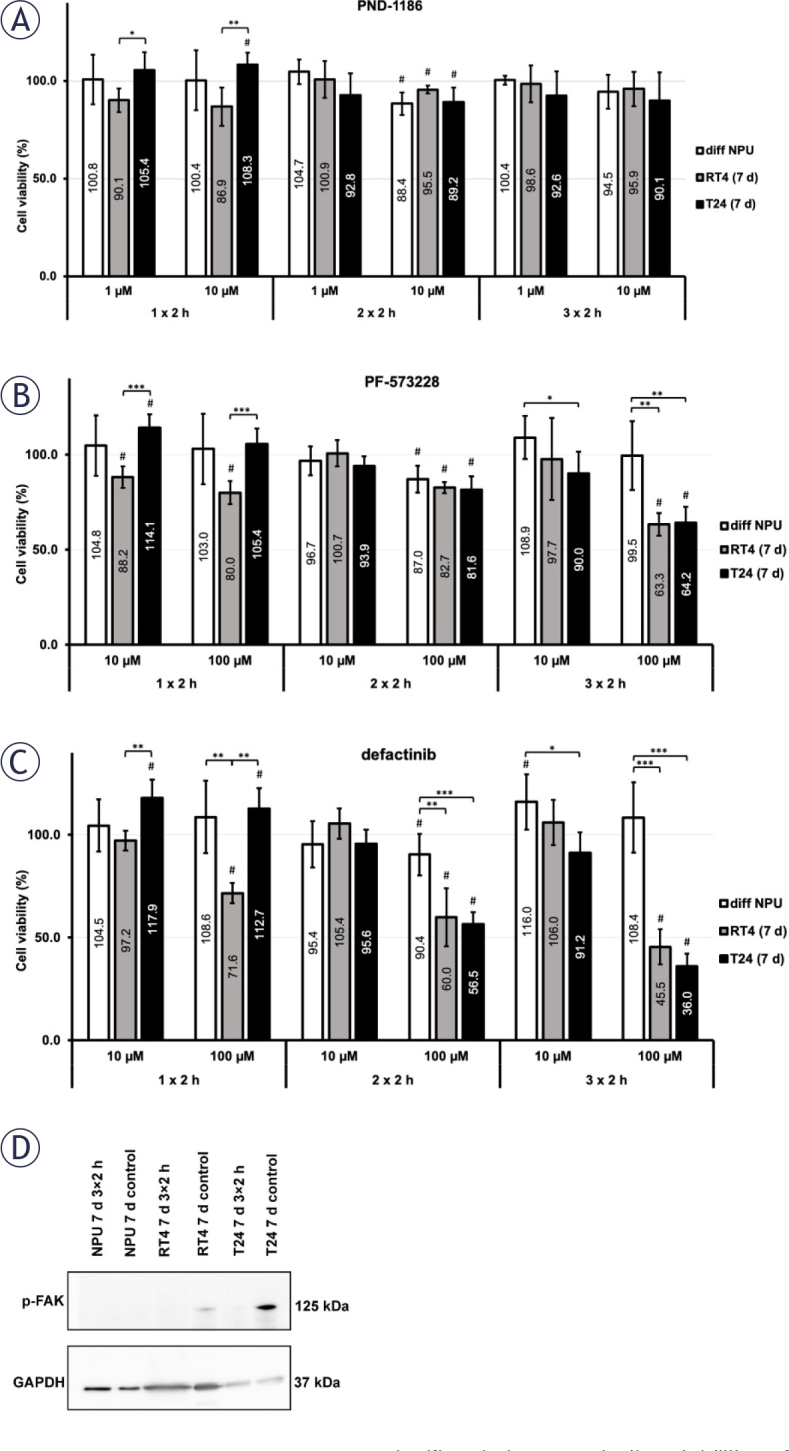
Effect of focal adhesion kinase (FAK) inhibitors on viabilities of differentiated normal urothelial (NPU) cells (diff NPU), 7-day RT4 and 7-day T24 cells (RT4 (7 d) and T24 (7 d)). **(A)** After treatment with 10 μM PND-1186 for 2 h per day for 3 consecutive days (3 × 2 h), no statistically significant differences in cell viability were observed among diff NPU, RT4 (7 d) and T24 (7 d). **(B)** Treatment with 100 μM PF-573228 for 3 × 2 h resulted in a significant decrease in the viabilities of both RT4 (7 d) and T24 (7 d) compared to the viability of diff NPU. **(C)** Treatment with 100 μM defactinib for 3 × 2 h caused the greatest difference between the viabilities of diff NPU, RT4 (7 d) and T24 (7 d). **(D)** Western blot of p-FAK after the 3 × 2 h treatment with 100 μM defactinib in 7-day NPU cells (NPU (7 d)), RT4 (7 d) and T24 (7 d) with GAPDH-loading control. Data are presented as the mean ± the standard error of the mean (SEM). To differentiate the statistical analysis, we compare the viability between different cell types (*, **, ***) and the viability between the control and the treated sample within each cell type (#). #P < 0.05. *P < 0.05. **P < 0.005. ***P < 0.001.

The treatment of *in vitro* models representing the differentiated urothelium and later stages following tumour resection showed similar trends in results to those observed in the early post-surgery phase *in vitro* models. Treatment with the FAK inhibitor PND-1186 at both tested concentrations resulted in a statistically significant increase in the viabilities of 7-day T24 cells after the 1 × 2 h treatment (105.4 ± 9.4% and 108.3 ± 6.3%). Incubation with 10 μM PND-1186 caused a statistically significant decrease in the viabilities of differentiated NPU, 7-day RT4 and 7-day T24 cells compared to their untreated controls after the 2 × 2 h treatment (88.4 ± 5.8%, 95.5 ± 2.0% and 89.2 ± 7.4%, respectively). A 3 × 2 h treatment did not result in significant changes in cell viabilities. (Figure 6A).

Treatment with 10 μM PF-573228 for 1 × 2 h led to a significantly higher viability of 7-day T24 (114.1 ± 7.0%) compared to the viability of 7-day RT4 cells (88.2 ± 5.6%). However, it led to a significantly lower viability of 7-day T24 (90.0 ± 11.4%) compared to differentiated NPU cells (108.9 ± 11.3%) after treatment for 3 × 2 h. Incubation with 100 μM PF-573228 caused a decrease in the viabilities of differentiated NPU, 7-day RT4 and 7-day T24 cells compared to their untreated controls after the 2 × 2 h treatment, but no significant differences between the viabilities of the different *in vitro* models (87.0 ±7.1%, 82.7 ± 2.8% and 81.6 ± 6.9%). A 3 × 2 h treatment resulted in a statistically significant reduction in the viabilities of 7-day RT4 and 7-day T24 cells (63.3 ± 6.1% and 64.2 ± 8.5%, respectively). In contrast, the viability of differentiated NPU cells remained high and was not significantly lower than that of the untreated control (99.5 ± 18.1%) (Figure 6B).

Again, defactinib was the most potent FAK inhibitor. Treatment with 10 μM defactinib caused, similarly to 10 μM PF-573228, a significantly higher viability of 7-day T24 (117.9 ± 8.9%) compared to 7-day RT4 cells (97.2 ± 4.7%) after 1 × 2 h of treatment and a significantly lower viability of 7-day T24 (91.2 ± 9.9%) compared to differentiated NPU cells (116.0 ± 13.4%) after 3 × 2 h of treatment. Incubation with 100 μM defactinib caused the greatest decrease in the viabilities of 7-day RT4 and 7-day T24 cells (45.5 ± 8.5% and 36.0 ± 6.2%, respectively) after the 3 × 2 h treatment, while differentiated NPU cells remained intact (108.4 ± 17.1%) (Figures 6C and Supplementary Figure 7). We additionally performed a western blot, which showed that p-FAK was present in 7-day RT4 and 7-day T24 cells without treatment but was absent after 3 × 2 h of treatment with 100 μM defactinib (Figure 6D). Supplementary Figure 8 illustrates the effect of FAK inhibitors on FAK and p-FAK expression in 7-day T24 cells after 24-hour treatment with FAK inhibitors. The p-FAK was not detected in either control or treated 7-day NPU cells, consistent with the resistance of NPU cells and the susceptibility of urothelial cancer cells to FAK inhibition observed in the cell viability assays.

### FAK inhibition leads to caspase-3-mediated apoptosis of urothelial cancer cells RT4 and T24

**FIGURE 7.**
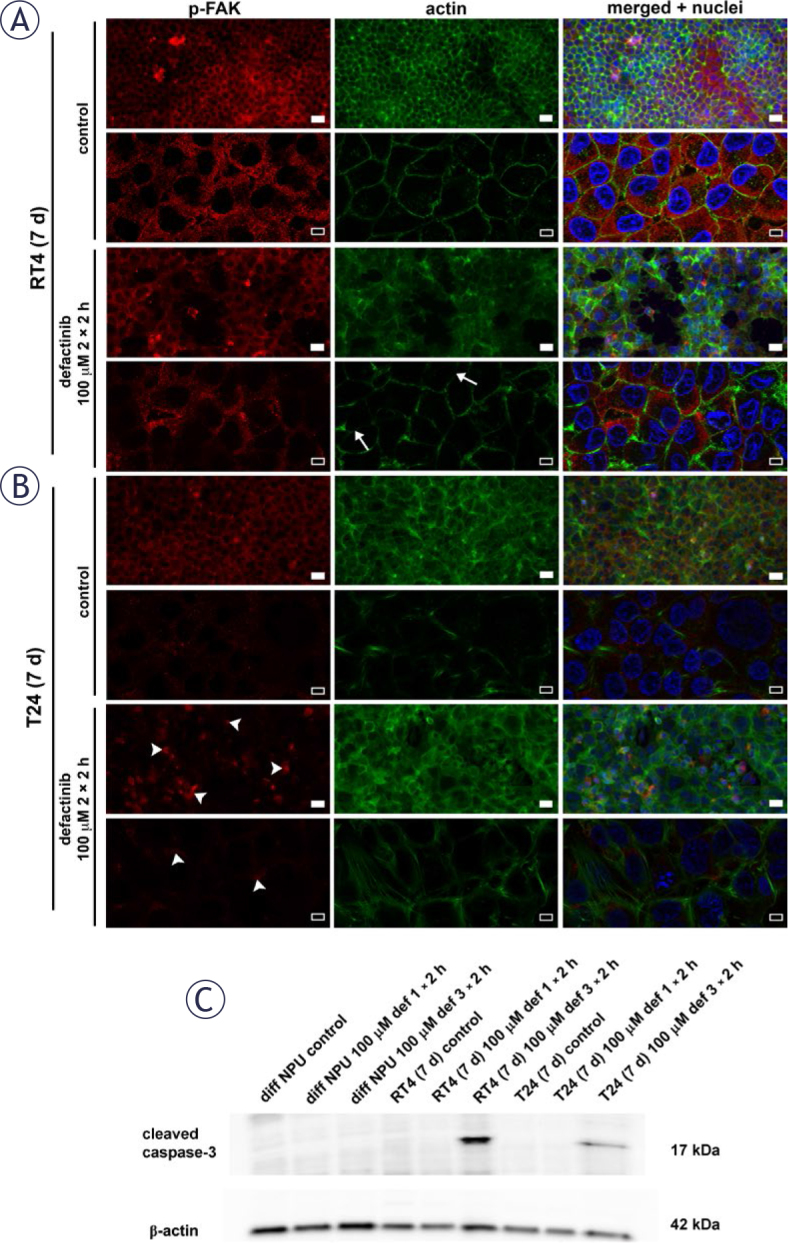
Focal adhesion kinase (FAK) inhibition with 100 μM defactinib for 2 h per day for 2 consecutive days (2 × 2 h) leads to caspase-3-mediated apoptosis. **(A)** Treated 7-day RT4 cells (RT4 (7 d)) showed lower p-FAK-labelling than controls. White arrows indicate gaps in the distribution of actin filaments in treated RT4 cells. **(B)** 7-day T24 cells (T24 (7 d)) contained p-FAK labelling in clusters after treatment with 100 μM defactinib for 2 × 2 h (white arrowheads). No significant changes in actin filament localisation were observed in treated and control T24 cells. Scale bars: 50 μm (white), 10 μm (black). **(C)** Western blot analysis showed that cleaved caspase-3 was present only in RT4 (7 d) and T24 (7 d) after treatment with 100 μM defactinib for 2 h per day for 3 consecutive days (3 × 2 h).

To visualise the effects of defactinib on urothelial cancer cells, we immunolabelled 7-day RT4, 7-day T24 and their control cells after the treatment with 100 μM defactinib for 2 × 2 h (this treatment regimen was chosen to obtain sufficient numbers of cells for analysis). After the treatment of 7-day RT4 cells for 2 × 2 h, we observed fewer p-FAK-labelled cells and no significant changes in the actin filament organisation compared with untreated cells (Figure 7A). After the treatment of 7-day T24 cells for 2 × 2 h, the clusters of p-FAK were observed, however no significant changes in the organisation of actin filaments were observed in comparison to untreated cells (Figure 7B).

To define the mechanism of action of FAK inhibitors, we analysed the type of cell death of normal urothelial and urothelial cancer cells. We performed a western blot of cleaved caspase-3, i.e. the large fragment (17/19 kDa) of activated cas-pase-3 produced by cleavage near Asp175 during apoptosis. Analysis showed cleaved caspase-3 in 7-day RT4 and 7-day T24 cells after 3 × 2 h of treatment with 100 μM defactinib. Cleaved caspase-3 was absent in all other treated *in vitro* models, including differentiated NPU cells, after 3 × 2 h of treatment with 100 μM defactinib, consistent with the results of cell viability assays (Figures 7C and Supplementary Figure 9).

## Discussion

FAK is a protein involved in cell adhesion, migration, and signalling pathways related to tumour growth and metastasis.^26^ Although not encoded by an oncogene, FAK overexpression and phosphorylation have been linked to several cancers, including squamous cell carcinoma of the head and neck^27^, neuroblastoma^28^, breast cancer^29^, ovarian cancer^30^ and colorectal cancer.^29^ In recent years, studies have shown that increased FAK expression is associated with poor prognosis in bladder cancer, highlighting FAK as a potential drug target.^13,14^

The aim of our study was to investigate the role of FAK in urothelial cancer cells, with a particular focus on how it differs from its role in normal urothelial cells *ex vivo* and *in vitro*. To this end, we analysed the expression levels of non-phosphorylated FAK and p-FAK in human bladder cancer tissue and normal bladder tissue biopsies. Our western blot analysis and immunohistochemical staining showed that the protein expression levels of FAK and p-FAK are higher in bladder cancer tissue than in normal tissue. These findings are consistent with those of Zhang *et al*, who showed that FAK expression is significantly higher in bladder cancer tissue compared to healthy tissue.^14^ However, in contrast to their findings, our study showed that the expression of FAK and p-FAK was not related to tumour stage and grade. Specifically, we found no differences in the total protein expression levels of FAK and p-FAK among pT1 LG, pT1 HG and pT2 HG tumours. This discrepancy in results may be due to the differences in the methods used to assess FAK expression. Zhang *et al*. performed immunohistochemical analysis and calculated the staining index based on the intensity and distribution of staining in a large number of samples.^14^ In contrast, we conducted western blot analysis on a smaller sample set (2–4 per tumour stage and grade) and performed immunohistochemistry on individual patient samples.

In addition to FAK and p-FAK, we investigated the expression of the adherens junctional proteins, E- and N-cadherin, in human bladder cancer and adjacent normal tissue biopsies. Cadherins are a family of cell adhesion molecules that play a critical role in maintaining tissue integrity and cell–cell interactions by regulating cell adhesion and migration.^31^ We showed that the expression of E- and N-cadherin was significantly deregulated in bladder cancer biopsies. Our study found that the expression levels of E- and N-cadherin were significantly higher in bladder cancer biopsies than in the adjacent healthy tissue. Furthermore, we found that the relationship between E- and N-cadherin expression was related to tumour stage and grade. Surprisingly, our results showed that E-cadherin expression is maintained in pT1 and pT2 stage tumours, with pT1 HG tumours expressing the highest protein level of E-cadherin. These results may be due to the high biological variability within bladder tumours, caused by various factors such as intratumoural heterogeneity and different molecular subtypes.^32–34^ The high expression of both E- and N-cadherin in pT1 and pT2 HG cancer tissue may reflect the complex and transitional nature of epithelial-to-mesenchymal transition (EMT) and its reversal, mesenchymal-to-epithelial transition (MET), in tumour progression. High-grade tumours often exhibit a hybrid epithelial/mesenchymal phenotype, where cells retain E-cadherin expression while also expressing N-cadherin, providing them with increased plasticity to balance adhesion and motility for local invasion.^35,36^ Furthermore, tumour heterogeneity plays a significant role, as the coexistence of epithelial and mesenchymal markers may indicate the presence of distinct subpopulations of cells at different stages of EMT or MET, reflecting the tumour’s dynamic nature.^37^ Aberrant activation of key signalling pathways, such as TGF-β or Wnt/β-catenin, can also upregulate both cadherins simultaneously, as these pathways are crucial regulators of EMT and are often dysregulated in aggressive cancers.^38^ Additionally, the co-expression of E- and N-cadherin may confer advantages in tumour survival and metastatic potential, enabling cells to dynamically adapt to various microenvironmental conditions during invasion and colonisation.^39^

Moreover, we have shown that N-cadherin expression is greater in pT1 and pT2 HG tumours compared with pT1 LG tumours, suggesting that N-cadherin expression increases with tumour stage and grade. N-cadherin is typically expressed in mesenchymal cells and its presence in high-grade tumours is often associated with poorer prognosis, increased metastatic potential and resistance to therapies.^40^ The upregulation of N-cadherin is often accompanied by the downregulation of E-cadherin, a process known as the cadherin switch, which is associated with tumour progression and metastasis.^41^ Our data support this claim, as we observed a slight decrease in E-cadherin expression in pT2 HG compared to pT1 HG tumours.

Following the analysis of focal adhesion and adherens junctional proteins in human biopsy samples, we have investigated and characterised the role of the above proteins in *in vitro* cell models of normal urothelium and bladder cancer. The significant differences in differentiation marker expression between normal urothelial cells and bladder cancer cells are well documented. Namely, normal urothelial cells represent a non-cancerous state characterised by high expression of uroplakins^42,43^, cytokeratins^44^ and E-cadherin^45^, while RT4 cells represent an early-stage bladder cancer phenotype, expressing markers associated with more differentiated or less aggressive bladder cancer cells, such as uroplakins^46^ and E-cadherin.^45^ On the other hand, T24 cells represent a more advanced stage of bladder cancer cells, characterised by increased invasiveness, the absence of differentiation markers (uroplakins)^47,48^ and upregulation of N-cadherin.^45,49^

In our experiment, we established a differentiated *in vitro* model of the urothelium (differentiated NPU cells) representing the normal bladder urothelium. Additionally, 2-day RT4 cells (papillary tumour model) and 2-day T24 cells (muscle-invasive tumour model) were used to simulate the residual urothelial cancer cells after resection of the bladder tumour. The 7-day RT4 and 7-day T24 cells, on the other hand, represented later stages after tumour resection. Our findings demonstrated that the urothelial differentiation markers *UPK1B* and *UPK3A* were highly expressed in differentiated NPU cells but were either downregulated or absent in 7-day RT4 and 7-day T24 cells, respectively. Furthermore, we showed that the expression of *CDH1*, the gene encoding E-cadherin, was higher in 7-day RT4 cells and differentiated NPU cells compared to 7-day T24 cells. In contrast, the expression of *CDH2*, the gene encoding N-cadherin, was highest in 7-day T24 cells. Our results were also confirmed by western blot analysis, providing further confirmation that the *in vitro* models used in our study can serve as valuable tools for understanding the disease and evaluating potential treatment options. Consistent with these findings, our previous study using urothelial *in vitro* models^45^ demonstrated that E-cadherin is more abundant in human non-invasive papilloma urothelial cell line RT4 compared to partially differentiated NPU, as shown by western blot analysis and fluorescent immunolabelling. In line with these results, the present study shows that 2-day RT4 cells exhibited the highest levels of E-cadherin on western blot. This could also help explain why pT1 HG samples showed the highest E-cadherin levels on western blot, while normal tissue adjacent to the analysed tumour samples displayed significantly lower or undetectable levels of E-cadherin. Additionally, our unpublished data demonstrate that E-cadherin is predominantly localized in the cytoplasm, rather than on the plasma membrane, of human muscle-invasive cancer urothelial cell line T24. This could also account for the elevated levels detected in the western blot analysis of pT1 HG and pT2 HG tumours.

Furthermore, we showed a significant increase in FAK expression and phosphorylation in 2-day and 7-day RT4 and T24 cells compared to 2-day and differentiated NPU cells. However, we did not detect any differences in FAK expression among the different bladder cancer cell lines using different molecular methods, indicating its potential role as a targeted molecular signalling pathway in the development of novel therapeutic interventions.

However, several practical questions remain unanswered regarding a potential treatment for bladder cancer targeting FAK. Key considerations include the optimal method for drug administration and the identification of drugs that can selectively eliminate bladder cancer cells while preserving the integrity of normal urothelial cells. These crucial aspects warrant further investigation to enhance our understanding and pave the way for more effective therapeutic strategies. To date, only a few studies have investigated the effect of FAK inhibition on urothelial cancer cells, while, to our knowledge, there are still no reports on the effects of FAK inhibition on normal urothelial cells. Using the T24 cell line, Kong *et al*. showed that both FAK silencing and inhibition of FAK phosphorylation by the FAK inhibitor PF-573228 suppressed bladder cancer cell invasion and migration and resulted in caspase-3-mediated apoptosis.^50,51^ Barlow *et al*. used organoids derived from RT4 cells and showed that the FAK inhibitor defactinib caused a dose-dependent decrease in FAK autophosphorylation and organoid size.^52^ In our study, we have demonstrated that FAK inhibition using specific inhibitors selectively targets bladder cancer cells, such as RT4 and T24, without affecting normal urothelial cells like NPU, confirming the potential for a targeted therapeutic approach.

We used two different approaches to inhibit FAK in our study. These included silencing FAK using miRNA and using specific FAK inhibitors PND-1186, PF-573228, and defactinib. We showed that FAK expression was only slightly reduced by miRNA silencing in RT4 cells, whereas the inhibition was significant in T24 cells. Our data also showed that all three plasmids induced significant apoptosis in both RT4 and T24 cells, with plasmids p44 and p45 specifically causing an increase in necrosis in T24 cells. Our observations align with previous reports in the literature, which show that downregulation of FAK using antisense oligonucleotides results in enhanced apoptosis in melanoma cell lines.^53^ While apoptosis has previously been linked to FAK downregulation or inhibition, the induction of necrosis following FAK downregulation represents a novel finding.^50^ In T24 cells, FAK downregulation was most pronounced with p45, followed by p46 and p44. Interestingly, only p44 and p45 were found to induce necrosis. This discrepancy may be attributed to the fact that mR-NA expression levels do not always correlate directly with protein expression levels.^54,55^ Moreover, the functional effects of FAK are influenced not only by its total protein levels but also by its phosphorylation status, which governs its biological activity. Since this part of our study evaluated FAK expression solely at the mRNA level, it is acknowledged as a limitation. Future studies incorporating protein quantification and analysis of FAK phosphorylation status, are warranted to provide a more comprehensive understanding of its role in the induction of necrosis.

Furthermore, we showed that treatment with 100 μM defactinib for 2 h per day for 3 consecutive days resulted in a significant reduction in the viabilities of RT4 and T24 cancer cells, while having no effect on normal urothelial cells. The treatment regimen of 2 h per day was chosen to align with clinical practice, where drugs used for intravesical treatment of bladder cancer are typically retained in the bladder for 2 h before voiding.^56^ None of the FAK inhibitors tested (the most effective concentrations were 10 μM PND-1186, 100 μM PF-573228 and 100 μM defactinib) caused a statistically significant decrease in the viability of differentiated urothelial cells after 3 days of treatment. However, defactinib was associated with the highest cell viability of differentiated urothelial cells and the lowest viability of urothelial cancer cells and was therefore identified as the FAK inhibitor with the greatest potential for further studies and as a treatment option following tumour resection in bladder cancer. Defactinib is currently being evaluated both as a single agent and in combination with other drugs in ongoing phase I and II clinical trials in ovarian cancer (NCT03287271, NCT05512208, NCT04625270, NCT02407509), pancreatic adenocarcinoma (NCT03727880, NCT04331041, NCT05669482), and other solid tumours.^57^

To better understand and further define the mechanism of action of FAK inhibitors, we analysed the mode of cell death in both normal urothelial and urothelial cancer cells. Increasing evidence suggests that FAK plays a crucial role in maintaining normal cell survival, with disruption of FAK signalling leading to a loss of substrate adhesion and anoikis (apoptosis) in anchorage-dependent cells, such as endothelial cells.^58^ We have demonstrated that FAK inhibition induced caspase-3-mediated apoptosis in urothelial cancer cells. It has been shown that in human endothelial cells, caspase-3 cleaves FAK at Asp-772 *in vitro*, generating a C-terminal FAK fragment, whose overexpression promotes the dephosphorylation of endogenous FAK.^59^ We hypothesise that, during the early stages of chemotherapy-induced apoptosis, caspase-3 cleaves FAK, generating the C-terminal fragment that has been shown to promote endogenous dephosphorylation of FAK, thereby further enhancing apoptosis. Therefore, FAK inhibition may prove to be even more effective when combined with chemotherapy.

The observed resistance of differentiated urothelium to all three FAK inhibitors may be due to its lower permeability compared to the increased permeability of much less differentiated cancer cells. In addition, we observed very low relative protein expression levels of FAK and p-FAK in differentiated NPU cells, which could further explain their resistance to FAK inhibitors. An effective blood-urine barrier depends on fully differentiated umbrella cells, which are characterised by the presence of urothelial plaques, slow turnover, limited growth potential and reduced endocytosis.^42,60,61^ Together with tight junctions, these characteristics make the blood-urine barrier the tightest and least permeable barrier in the human body.^10,61^ In our study, we observed significantly higher expression levels of *UPK1B* and *UPK3A* in differentiated NPU compared to 7-day RT4 and 7-day T24 cells. On the other hand, the loss of urothelial differentiation following the loss of umbrella cells is a critical event in the development and progression of bladder cancer.^62^ Thus, the blood-urine barrier in bladder cancer tissue exhibits very limited resistance, which has significant implications for treatment involving intravesical drug instillations. Using RT4 and T24 cells, Lojk *et al*. demonstrated that the efficacy of nanoparticle administration was significantly higher in urothelial cancer cells than normal urothelial cells, suggesting that the sensitivity of cells to drugs is largely dependent on their degree of differentiation.^11^

### Limitations of the study

The main limitation of our study arises from the limited number of human biological samples used in certain analyses (2–4 biopsies), which may introduce bias into the statistical outcomes. Larger sample sizes would improve the statistical power and robustness of the findings. Specifically, the analysis included three samples of pT1 tumours and two samples of pT2 tumours, which may explain the differences in E-cadherin levels. We hypothesise that, as T1 represents an earlier stage of tumour development, it might still benefit from E-cadherinmediated cell-to-cell junctions to facilitate tumour survival and angiogenesis. On the other hand, as the tumour advances and tight junctions disintegrate, E-cadherin may still be expressed and detected by antibodies, although it may no longer be functional at the plasma membrane. This could potentially explain the high levels of E-cadherin observed in pT1 tumours.

A limitation of our study is the absence of a control miRNA, which could help to delineate the non-specific effects of plasmid DNA delivery. It is known that plasmid DNA introduced into cells can activate DNA sensing pathways, potentially triggering immune responses or leading to cell death.^63,64^ Therefore, some portion of the cell death observed following gene electrotransfer (GET) of anti-FAK plasmids may be attributed to these non-specific effects. Despite this, our findings are supported by the induction of caspase-3-mediated apoptosis observed with FAK inhibitors, which directly target and suppress FAK activity. The similarity in outcomes between these approaches strongly suggests that the primary mechanism driving apoptosis in our study is the inhibition of FAK rather than the non-specific activation of DNA sensing pathways. To further validate this conclusion, future studies could incorporate a control miRNA to better distinguish specific effects from potential artefacts of plasmid delivery. Additionally, experiments using scrambled miRNA or an empty plasmid would further help confirm that the observed effects are due to FAK inhibition rather than off-target or plasmid-related artefacts.

Although we identified caspase-3-mediated apoptosis after FAK inhibition, more comprehensive analysis of the apoptotic pathway, including upstream and downstream regulators, would provide a deeper understanding of the molecular events leading to cell death.

## Conclusions

In our study, we investigated the molecular characteristics of bladder cancer tissues, focusing on the expression of E-cadherin, N-cadherin, FAK, and p-FAK. We observed higher expression levels of these proteins in *ex vivo* bladder cancer tissues compared to normal tissues, with a notable prevalence of FAK and p-FAK in high-grade cancer tissues. Similarly, in *in vitro* models, bladder cancer cell lines (RT4 and T24) exhibited significantly higher FAK expression than normal urothelial cells (NPU). We silenced FAK expression through gene electrotransfer, which led to apoptosis and necrosis specifically in cancer cells. Additionally, we examined the effects of various FAK inhibitors on cell viability. Among them, defactinib demonstrated the most promising results by significantly reducing cancer cell viability while sparing normal urothelial cells. We have also shown that this mechanism involves caspase-3-mediated apoptosis in cancer cells. Our findings highlight the potential of defactinib as a therapeutic agent for targeting residual urothelial cancer cells following bladder tumour resection, utilizing intravesical instillations – an established therapeutic approach. The insights from our study may enhance healthcare professionals’ understanding of the molecular complexities of bladder cancer and provide a foundation for the development of more targeted and effective treatments.

## Supplementary Material

Supplementary Material Details
