# Supplementary material for: The role of focal adhesion kinase in bladder cancer: translation from in vitro to ex vivo human urothelial carcinomas
Source: Radiol Oncol. 2025 Sep 5;59(3):349–67. doi: 10.2478/raon-2025-0052 (PMC12441887; doi:10.2478/raon-2025-0052)
Supplement: Supplementary file 1 — Supplementary Material Details [file raon-2025-0052_sm.pdf]

The role of focal adhesion kinase in bladder cancer: translation from *in vitro* to *ex vivo* human urothelial carcinomas

Gaja Markovic, Natasa Resnik, Aleksandar Janev, Dasa Zupancic, Gasper Grubelnik, Marusa Debeljak, Maja Cemazar, Tanja Jesenko, Masa Omerzel, Tomaz Smrkolj, Mateja Erdani Kreft

doi: 10.2478/raon-2025-0052

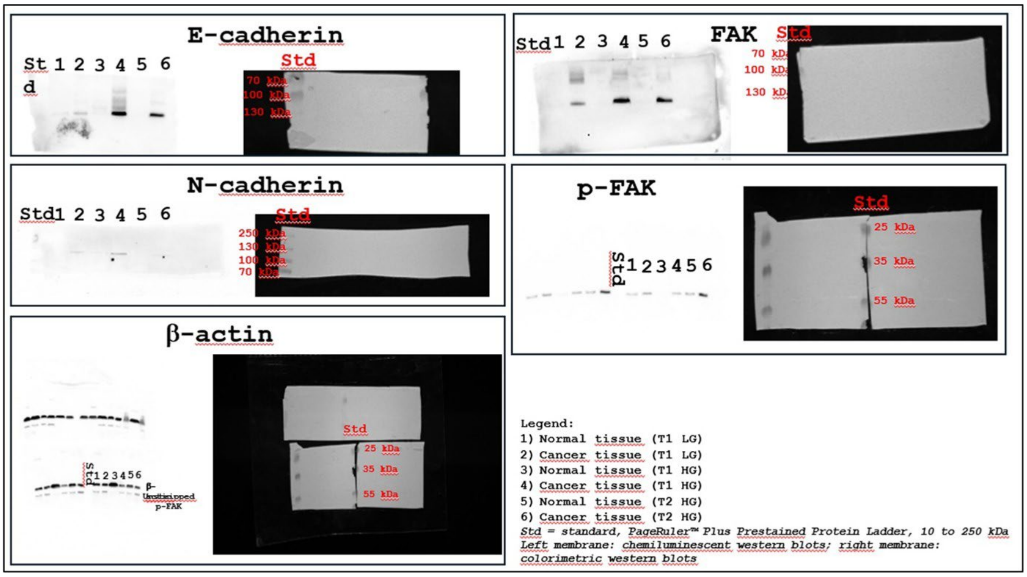

SUPPLEMENTARY FIGURE 1. Complete, unedited images of Western blot analysis of E-cadherin, N-cadherin, focal adhesion kinase (FAK), phosphorylated FAK (p-FAK), and β-actin in normal and cancerous tissues, provided as Supplementary images to Figure 2A. Results are shown for six samples: Normal tissue (T1 low-grade (LG)), Cancer tissue (T1 LG), Normal tissue (T1 high-grade (HG)), Cancer tissue (T1 HG), Normal tissue (T2 HG), and Cancer tissue (T2 HG). The left panels display chemiluminescent Western blots, while the right panels show colorimetric Western blots. A PageRuler™ Plus Prestained Protein Ladder (10 to 250 kDa) was used as the molecular weight reference, with molecular weight markers highlighted in red.

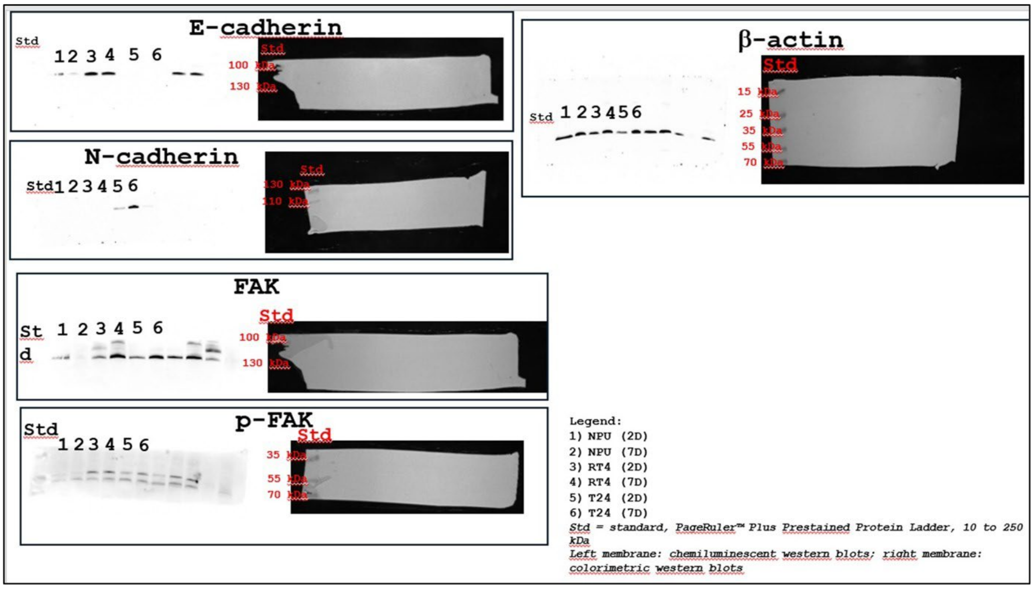

SUPPLEMENTARY FIGURE 2. Complete, unedited images of Western blot analysis of E-cadherin, N-cadherin, FAK, p-FAK, and β-actin in normal porcine urothelial (NPU), RT4, and T24 cells (2-days and 7-days), provided as supplementary images to Figure 3B. The left panels display chemiluminescent Western blots, while the right panels show colorimetric Western blots. A PageRuler™ Plus Prestained Protein Ladder (10 to 250 kDa) was used as the molecular weight reference, with molecular weight markers highlighted in red.

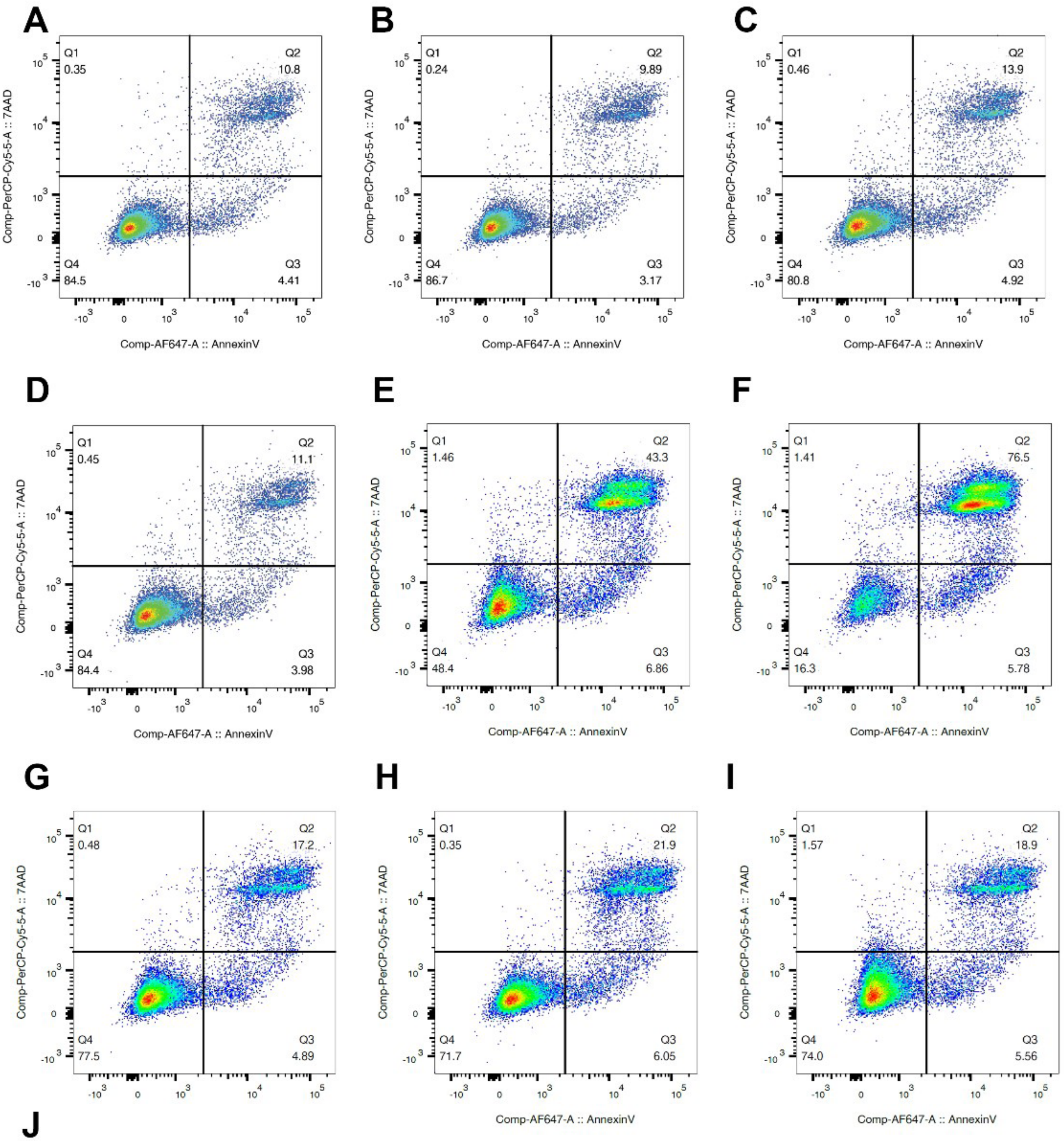

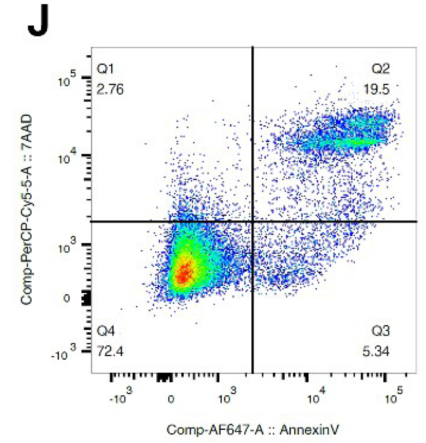

SUPPLEMENTARY FIGURE 3. Flow cytometry plots of first experiment in RT4 cells. X-axes represent Annexin V fluorescent signal and Y-axes 7-aminoactinomycin D (7-AAD) fluorescent signal. Live cells are identified as the subset of cells negative for both Annexin V and 7-AAD staining (Q4). Dead cells are defined as the subset negative for Annexin V and positive for 7-AAD staining (Q1). Apoptotic cells are determined as the sum of two subsets: early apoptotic cells, characterized by positive Annexin V and negative 7-AAD staining (Q3), and late apoptotic cells, characterized by dual positivity for Annexin V and 7-AAD (Q2). Samples are (A) Ctrl_1, (B) Ctrl_2, (C) EP_1, (D) EP_2, (E) p44_1, (F) p44_2, (G) p45_1, (H) p45_2, (I) p46_1, (J) p46_2.

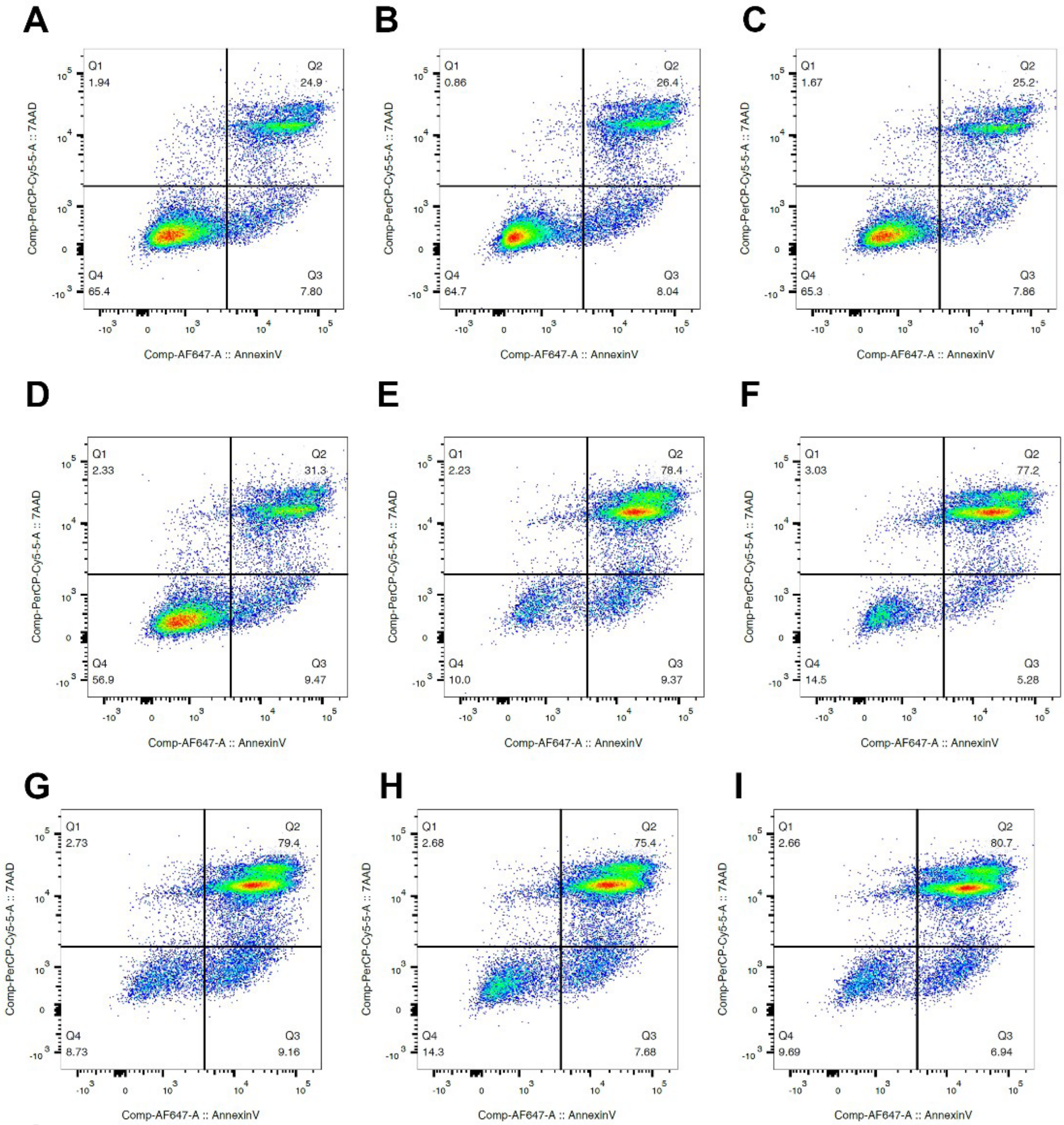

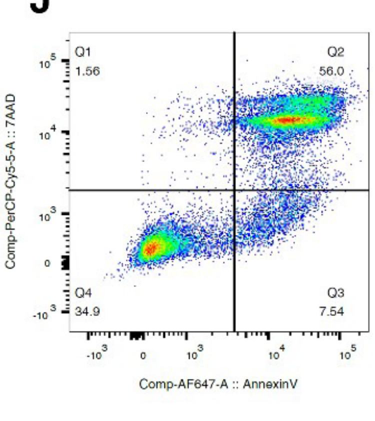

SUPPLEMENTARY FIGURE 4. Flow cytometry plots of second experiment in RT4 cells. X-axes represent Annexin V fluorescent signal and y-axes 7-AAD fluorescent signal. Live cells are identified as the subset of cells negative for both Annexin V and 7-AAD staining (Q4). Dead cells are defined as the subset negative for Annexin V and positive for 7-AAD (Q1). Apoptotic cells are determined as the sum of two subsets: early apoptotic cells, characterized by positive Annexin V and negative 7-AAD staining (Q3), and late apoptotic cells, characterized by dual positivity for Annexin V and 7-AAD (Q2). Samples are (A) Ctrl_1, (B) Ctrl_2, (C) EP_1, (D) EP_2, (E) p44_1, (F) p44_2, (G) p45_1, (H) p45_2, (I) p46_1, (J) p46_2.

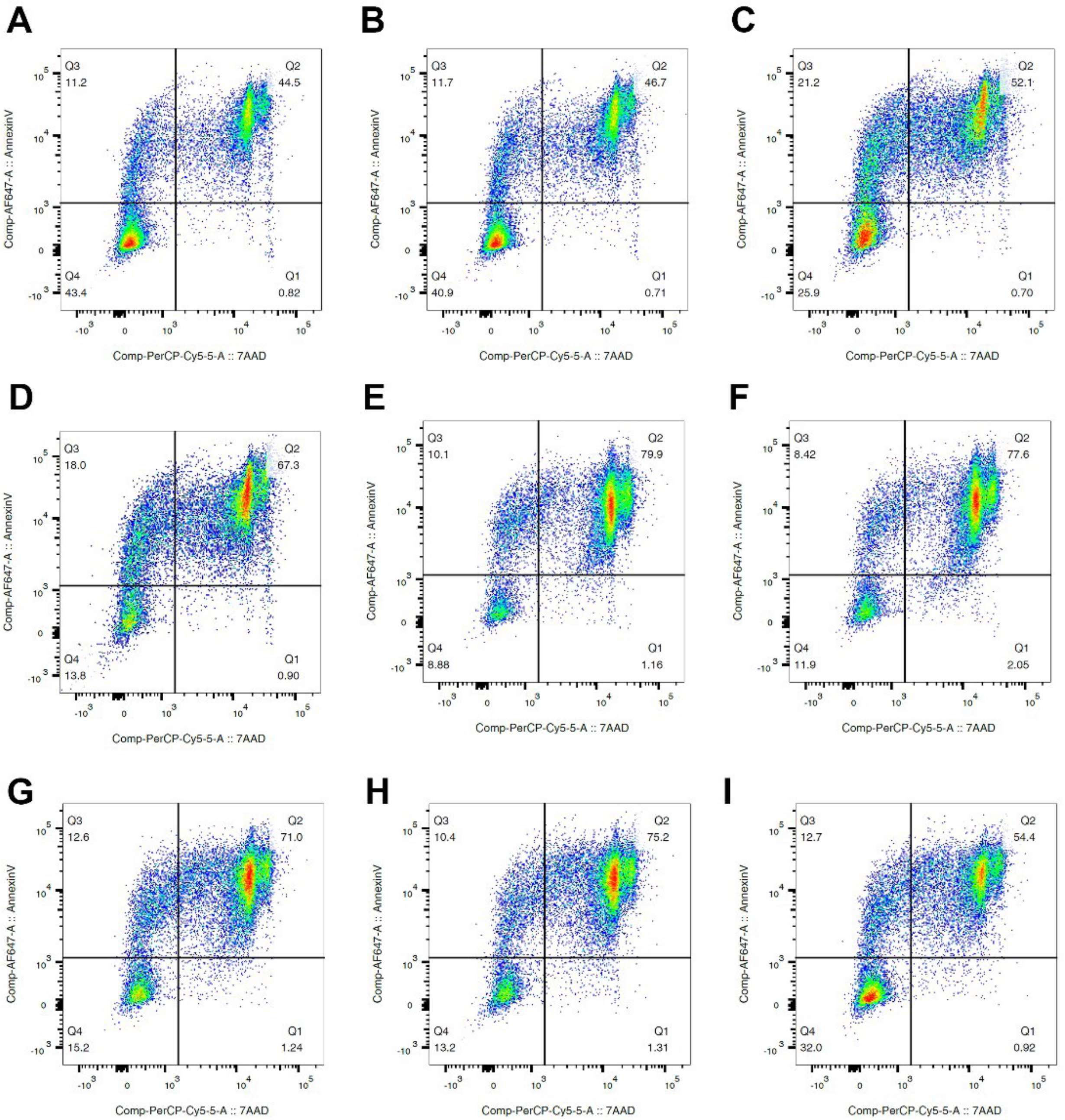

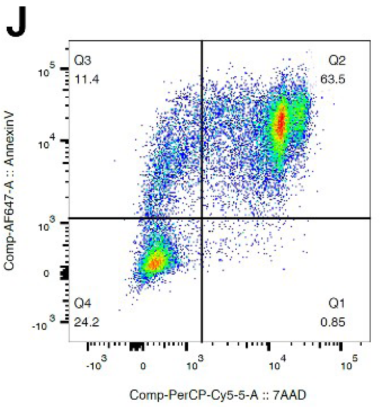

SUPPLEMENTARY FIGURE 5. Flow cytometry plots of first experiment in T24 cells. X-axes represent Annexin V fluorescent signal and y-axes 7-AAD fluorescent signal. Live cells are identified as the subset of cells negative for both Annexin V and 7-AAD staining (Q4). Dead cells are defined as the subset negative for Annexin V and positive for 7-AAD staining (Q1). Apoptotic cells are determined as the sum of two subsets: early apoptotic cells, characterized by positive Annexin V and negative 7-AAD staining (Q3), and late apoptotic cells, characterized by dual positivity for Annexin V and 7-AAD (Q2). Samples are (A) Ctrl_1, (B) Ctrl_2, (C) EP_1, (D) EP_2, (E) p44_1, (F) p44_2, (G) p45_1, (H) p45_2, (I) p46_1, (J) p46_2.

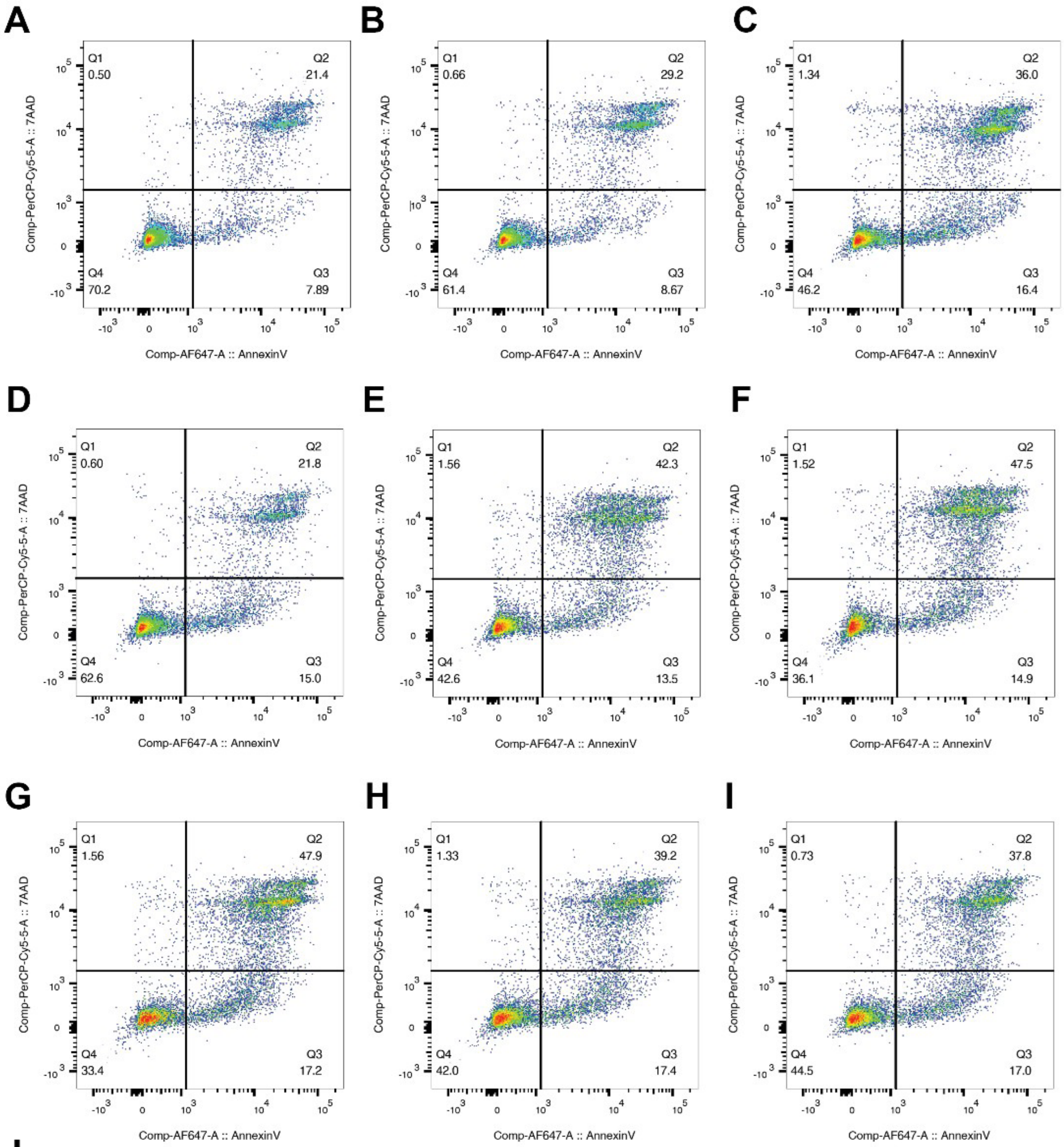

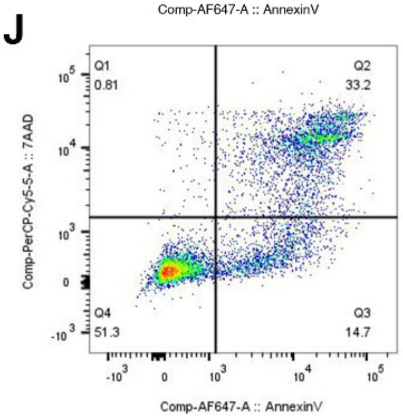

SUPPLEMENTARY FIGURE 6. Flow cytometry plots of second experiment in T24 cells. X-axes represent Annexin V fluorescent signal and y-axes 7-AAD fluorescent signal. Live cells are identified as the subset of cells negative for both Annexin V and 7-AAD staining (Q4). Dead cells are defined as the subset negative for Annexin V and positive for 7-AAD staining (Q1). Apoptotic cells are determined as the sum of two subsets: early apoptotic cells, characterized by positive Annexin V and negative 7-AAD staining (Q3), and late apoptotic cells, characterized by dual positivity for Annexin V and 7-AAD (Q2). Samples are (A) Ctrl_1, (B) Ctrl_2, (C) EP_1, (D) EP_2, (E) p44_1, (F) p44_2, (G) p45_1, (H) p45_2, (I) p46_1, (J) p46_2.

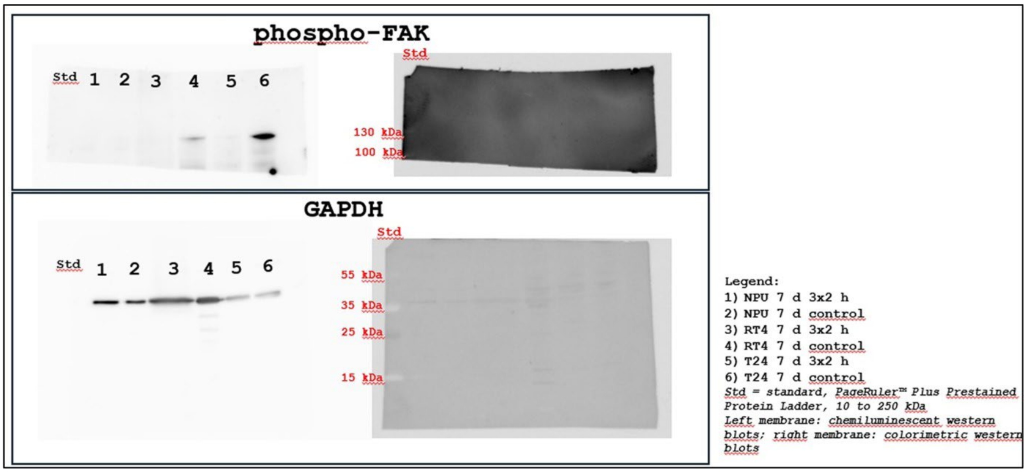

SUPPLEMENTARY FIGURE 7. Complete, unedited images of Western blot analysis of p-FAK and glyceraldehyde -3-phosphate dehydrogenase (GAPDH) in NPU, RT4, and T24 cells (7D, 3×2 h treatment vs. control), provided as supplementary images to Figure 6C. The left panels display chemiluminescent Western blots, while the right panels show colorimetric Western blots. A PageRuler™ Plus Prestained Protein Ladder (10 to 250 kDa) was used as the molecular weight reference, with molecular weight markers highlighted in red.

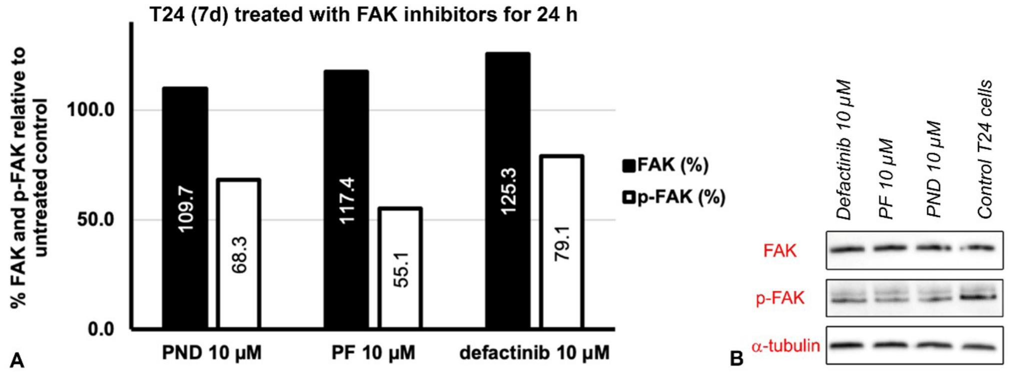

SUPPLEMENTARY FIGURE 8. (A) The graph illustrates the effect of FAK inhibitors on FAK and p-FAK expression in the T24 cell line after 7 days (7d) of cultivation and 24-hour treatment with FAK inhibitors. The y-axis represents the percentage of FAK and p-FAK expression relative to untreated control cells. Black bars indicate total FAK levels (%), while white bars represent p-FAK levels (%). (B) Western blot (WB) results further demonstrate the effect of FAK inhibitors on FAK and p-FAK expression. WB analysis was performed on T24 cells treated for 24 hours (on day 7 of cultivation) with FAK inhibitors or cultured in medium alone (control). The results confirm a reduction in p-FAK levels following treatment with FAK inhibitors. This analysis is based on one biological replicate with two technical replicates. The tested compounds included PND-1186 (PND, 10 µM), PF-573228 (PF, 10 µM), and defactinib (10 µM). The data suggest that FAK inhibitors reduce p-FAK levels, while total FAK levels remain unchanged or slightly increased.

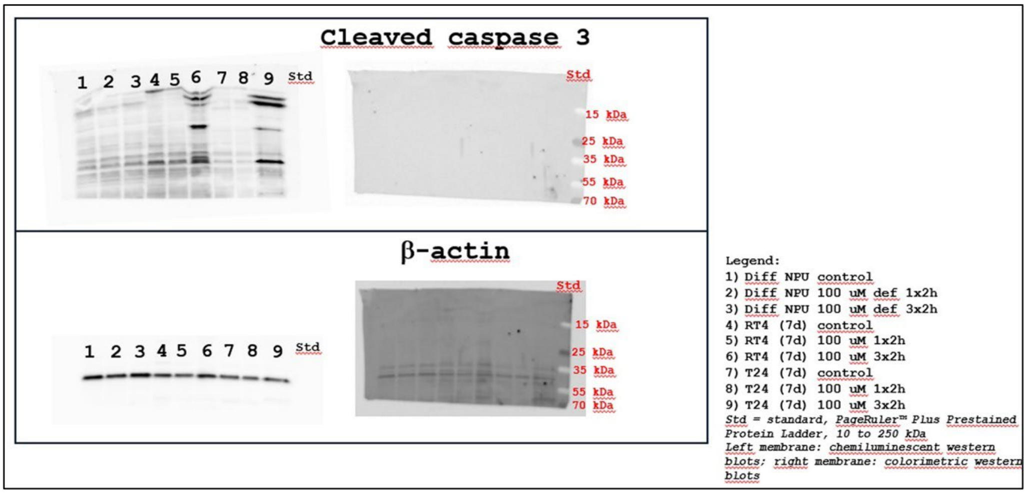

SUPPLEMENTARY FIGURE 9. Complete, unedited images of Western blot analysis of cleaved caspase-3 and β-actin in NPU, RT4, and T24 cells (control vs. 100 μM defactinib treatment: 1×2 h and 3×2 h), provided as supplementary images to Figure 7C. The left panels display chemiluminescent Western blots, while the right panels show colorimetric Western blots. A PageRuler™ Plus Prestained Protein Ladder (10 to 250 kDa) was used as the molecular weight reference, with molecular weight markers highlighted in red.
